# Design and In Vitro Evaluation of Splice-Switching Oligonucleotides Bearing Locked Nucleic Acids, Amido-Bridged Nucleic Acids, and Guanidine-Bridged Nucleic Acids

**DOI:** 10.3390/ijms22073526

**Published:** 2021-03-29

**Authors:** Takenori Shimo, Yusuke Nakatsuji, Keisuke Tachibana, Satoshi Obika

**Affiliations:** Graduate School of Pharmaceutical Sciences, Osaka University, 1-6 Yamadaoka, Suita, Osaka 565-0871, Japan; shimo-t@phs.osaka-u.ac.jp (T.S.); nktj101@outlook.jp (Y.N.); nya@phs.osaka-u.ac.jp (K.T.)

**Keywords:** exon skipping, splice-switching oligonucleotide, chemical modification, bridged nucleic acids (BNAs), locked nucleic acids (LNAs), amido-bridged nucleic acids (AmNAs), guanidine-bridged nucleic acids (GuNAs), dystrophin

## Abstract

Our group previously developed a series of bridged nucleic acids (BNAs), including locked nucleic acids (LNAs), amido-bridged nucleic acids (AmNAs), and guanidine-bridged nucleic acids (GuNAs), to impart specific characteristics to oligonucleotides such as high-affinity binding and enhanced enzymatic resistance. In this study, we designed a series of LNA-, AmNA-, and GuNA-modified splice-switching oligonucleotides (SSOs) with different lengths and content modifications. We measured the melting temperature (*T*_m_) of each designed SSO to investigate its binding affinity for RNA strands. We also investigated whether the single-stranded SSOs formed secondary structures using UV melting analysis without complementary RNA. As a result, the AmNA-modified SSOs showed almost the same *T*_m_ values as the LNA-modified SSOs, with decreased secondary structure formation in the former. In contrast, the GuNA-modified SSOs showed slightly lower *T*_m_ values than the LNA-modified SSOs, with no inhibition of secondary structures. We also evaluated the exon skipping activities of the BNAs in vitro at both the mRNA and protein expression levels. We found that both AmNA-modified SSOs and GuNA-modified SSOs showed higher exon skipping activities than LNA-modified SSOs but each class must be appropriately designed in terms of length and modification content.

## 1. Introduction

Splicing modulation is becoming a therapeutic strategy for many genetic diseases. At present, the US Food and Drug Administration (FDA) has approved the splice-switching oligonucleotide (SSO) therapeutic drugs eteplirsen, golodirsen, and viltolarsen for the treatment of Duchenne muscular dystrophy and nusinersen for the treatment of spinal muscular atrophy [1]. Additionally, many SSOs are currently undergoing clinical trials [2]. However, only a few chemical modifications have been utilized in the FDA-approved SSOs [3]. Specifically, eteplirsen, golodirsen, and viltolarsen are phosphorodiamidate morpholino oligomers (PMOs), and nusinersen is a 2′-*O*-methoxyethyl RNA (2′-MOE RNA) with phosphorothioate modifications. Of course, both types of chemical modifications have been well studied for splicing modulation [4], but there are many additional chemistries that have been investigated for SSOs. Many research groups, including ours, have proposed chemical modifications to improve RNA binding affinity as an approach to increase the splicing modulation efficiency of SSOs [5,6,7,8,9,10,11,12,13]. In addition, recent studies have focused on other properties of SSO chemical modifications, such as the promotion of splicing factor recruitment and inhibition of secondary structure formation [6,14]. In 2012, Rigo et al. reported that introduction of 2′-deoxy-2′-fluoro-RNA (2′-F RNA) into SSOs led to the recruitment of the interleukin enhancer binding factor 2 and 3 complex [14,15]. We also reported that the formation of higher-order structures in SSOs was suppressed and exon skipping efficiency was increased by introducing 7-deaza-2-deoxyguanosine or 2′-deoxyinosine into SSOs [6]. Thus, ongoing investigations of the characteristics of chemical modifications for efficient splicing modulation are important.

**Figure 1:**
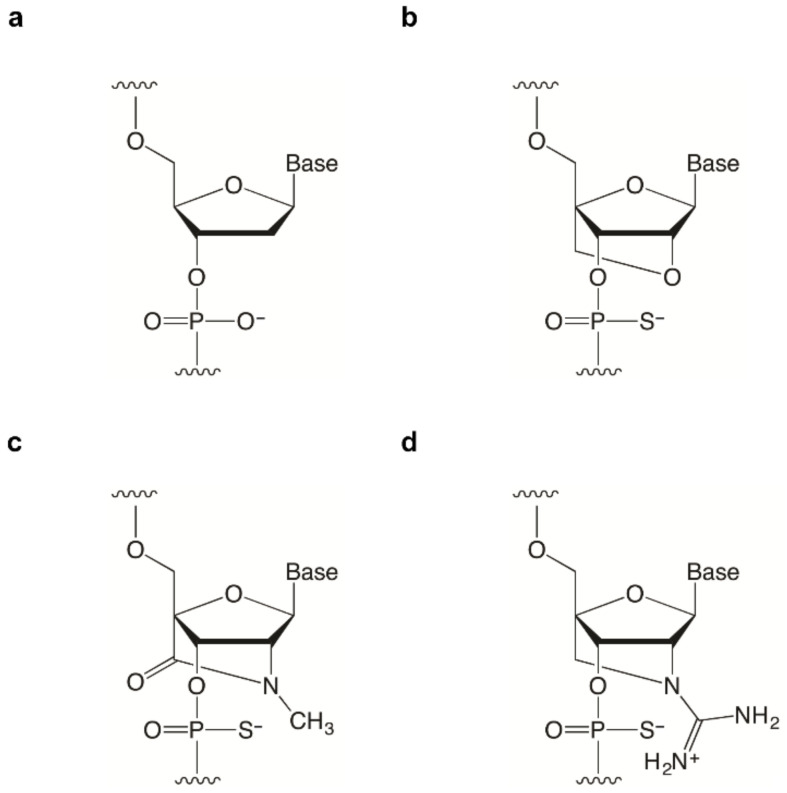
Structures of BNA analogs used in this study. (**a**) Deoxyribonucleic acid (DNA). (**b**) 2′, 4′-Bridged nucleic acid/locked nucleic acid (2′,4′-BNA/LNA). (**c**) Amido-bridged nucleic acid (AmNA). (**d**) Guanidine-bridged nucleic acid (GuNA).

To date, our group has developed a series of bridged nucleic acids (BNAs), including amido-bridged nucleic acid (AmNA) and guanidine-bridged nucleic acid (GuNA) (Figure 1) [16,17]. The RNA binding affinity of oligonucleotides modified with AmNA or GuNA is very high, similar to that of locked nucleic acid (LNA) oligonucleotides [16,17]. In addition, the nuclease resistance of oligonucleotides modified with AmNA or GuNA is more than 10 times better than that of LNA-modified oligonucleotides [16,17]. Although we have reported that AmNA-modified gapmers are effective in suppressing targeted gene expression in vivo, the potential application of AmNA and GuNA as SSOs to regulate splicing has not yet been investigated. Therefore, we have performed experiments using SSOs modified with AmNA and GuNA to clarify whether these bridged nucleic acids are able to modulate splicing. When incorporating chemical modification into SSOs, it is necessary to consider optimal designs, such as the SSO length and content of chemical modifications. Other groups have already reported the optimal designs of SSOs for PMOs and 2′-*O*-methyl (2′-OMe) RNA [18,19,20]. In contrast, other group and ours found that the optimal designs of LNA-modified SSOs are completely different from those of PMO- and 2′-OMe RNA-modified SSOs [5,11,21,22,23]. Thus, we predicted that optimized AmNA and GuNA would also have unique designs.

In this study, we tested this prediction by designing a series of LNA-, AmNA-, and GuNA-modified SSOs that target human *DMD* (*Dystrophin*) exons 58 and 50 and investigating whether both AmNA- and GuNA-modified SSOs could induce exon skipping in vitro. We also revealed that different designs are necessary for LNA-, AmNA-, and GuNA-modified SSOs to obtain efficient exon skipping and splicing modulation.

## 2. Results

### 2.1. Design of BNA-Modified SSOs Targeting DMD Exon 58 and Evaluation Using a Stably Transfected Cell Line

**Figure 2:**
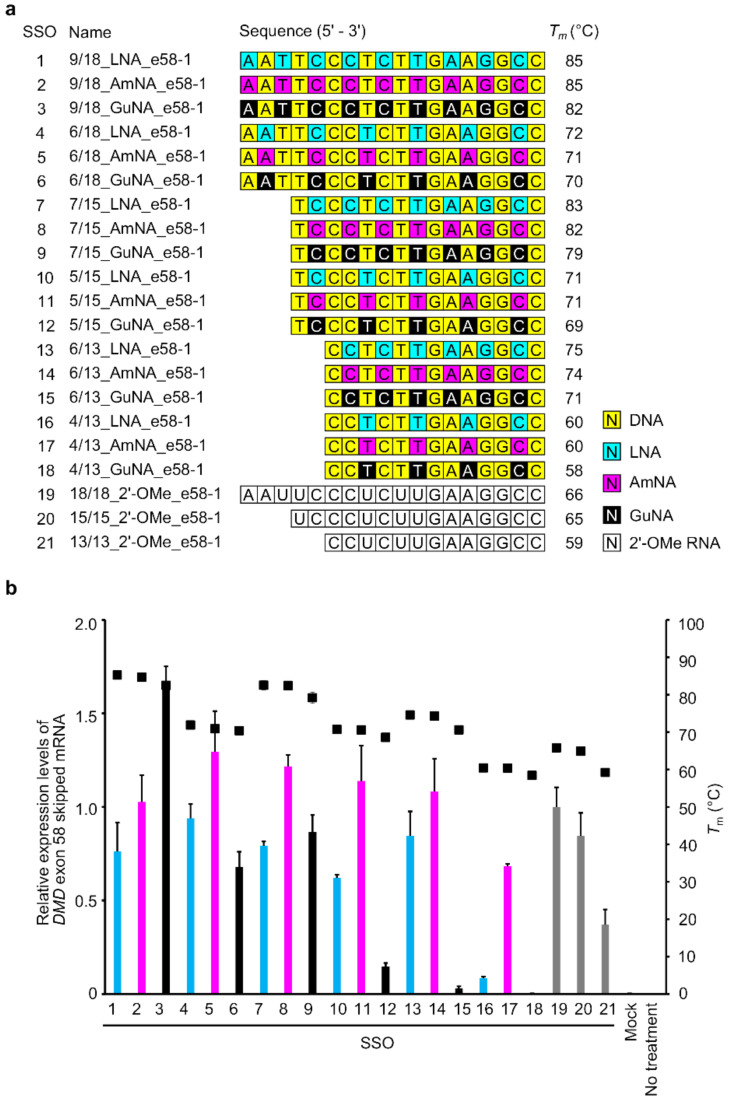
Design and evaluation of 13- to 18-mer LNA/AmNA/GuNA/2′-OMe RNA-modified splice-switching oligonucleotides (SSOs) targeting *DMD* exon 58 and having different lengths and modification content. (**a**) Schematic representation of 13- to 18-mer LNA, AmNA, GuNA, and 2′-OMe RNA-modified SSOs targeting *DMD* exon 58 designed for the assay. (**b**) Evaluation of 13- to 18-mer BNA-modified SSOs targeting *DMD* exon 58 and having different lengths and modification content using the stably transfected cells, stable-e58. Results of RT-qPCR analysis. The stable-e58 cells were transfected with the indicated SSOs (10 nM) for 24 h. On the *X*-axis of the graph, transfected SSOs are shown with SSO numbers mentioned in (**a**). The expression of exon 58 skipped mRNA was measured by RT-qPCR (bar). The expression was normalized to the *RPLP2* mRNA signal, relative to the value of 18/18_2′-OMe_e58-1 (SSO19) set to 1. Values represent the mean ± standard deviation of three independent experiments performed in duplicate. The melting temperature (*T*_m_) value of SSOs was measured from 5 to 94.5 °C (black box). Mock: treated with Lipofectamine only; no treatment: no transfection. Values represent the mean ± standard deviation of three independent experiments.

We previously reported that LNA-modified SSOs have both optimal LNA contents and appropriate lengths [5,21]. In the case of targeting *DMD* exon58, LNA-modified SSOs with the length of 7-mer to 23-mer showed exon skipping activity; among them, 13-mer and 15-mer SSOs were more effective. On the other hand, longer LNA-modified SSOs showed lower exon skipping activity, contrary to our expectation, because the longer LNA-modified SSOs themselves formed a secondary structure [6]. To investigate the ability of AmNA- and GuNA-modified SSOs to modulate splicing, we designed a series of SSOs of different lengths (13-, 15-, and 18-mers) targeting *DMD* exon 58 and possessing different contents of LNA, AmNA, and GuNA (Figure 2a and Supplementary Table S1).

First, we measured the melting temperature (*T*_m_) of each designed SSO when annealed to its complementary RNA strand (Figure 2a and Supplementary Table S2). Compared with the LNA-modified SSOs, the AmNA-modified SSOs showed almost the same or slightly lower RNA binding ability; the *T*_m_ values of the AmNA-modified SSOs were up to 1 °C lower than those of the LNA-modified SSOs. In contrast, the *T*_m_ values of the GuNA-modified SSOs were 1–4 °C lower than those of the LNA-modified SSOs. In particular, the GuNA-modified SSOs with many GuNA-modifications (9/18_GuNA_e58-1 (SSO3), 7/15_GuNA_e58-1 (SSO9), and 6/13_GuNA_e58-1 (SSO15)) showed a decrease in *T*_m_ of more than 2 °C than the corresponding LNA-modified SSOs. Although our previous studies revealed that oligonucleotides containing GuNA do not show a reduction in *T*_m_ [17], the results herein suggest that GuNA modifications to mixed antisense oligonucleotides increase binding affinity to the target RNA, although the effect may be less than that of the corresponding LNA modification.

**Figure 3:**
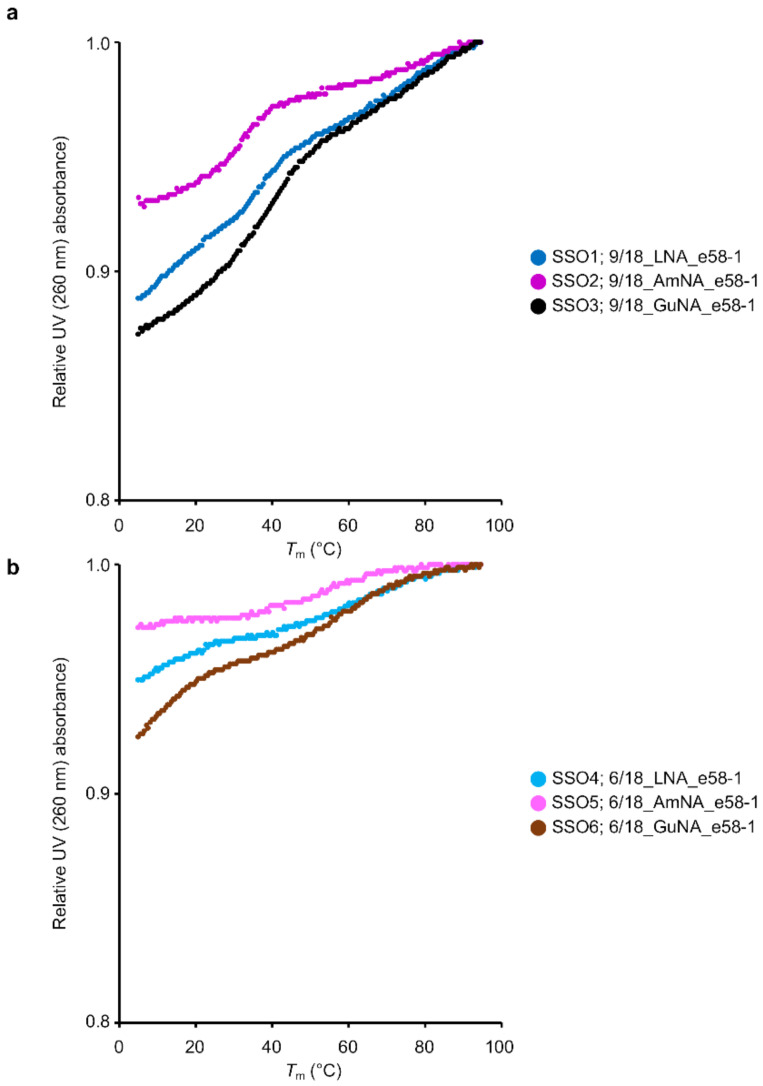
UV melting analysis of 18-mer LNA/AmNA/GuNA-modified SSOs targeting *DMD* exon 58 and having different modification content. (**a**) The result of UV melting experiments for single-stranded LNA-, AmNA-, and GuNA-modified SSOs having high modification content. (**b**) The result of UV melting experiments for single-stranded LNA-, AmNA-, and GuNA-modified SSOs having low modification content. All analyses were repeated three times to ensure reproducibility (see Supplementary Figure S1). The relative UV absorbance depicts normalized data (normalized by UV absorption at 94.5 °C set to 1).

In our previous study, we showed that longer LNA-modified SSOs form a secondary structure, which results in a decrease in exon skipping activity [6]. Therefore, we investigated whether the SSOs synthesized in the current study also formed secondary structures. Native polyacrylamide gel electrophoresis and ultraviolet (UV) melting analysis of SSOs in the absence of complementary strands are useful to confirm the formation of secondary structures [6]. In this study, we used the latter approach (Figure 3 and Supplementary Figure S1). If the SSO forms a secondary structure, a significant change in UV absorbance is observed as the temperature increases. The degree of change in UV absorbance (hyperchromic effects) and the temperature at which the change in UV absorbance was observed (*T*_m-ssSSO_) were considered indicators to determine whether secondary structures formed. In this report, the difference between the absorbance at 260 nm at 5 °C and that at 94.5 °C will be referred to as the hyperchromic effects. As shown in Figure 3, the 18-mer AmNA-modified SSOs clearly showed lower hyperchromic effects compared with the corresponding LNA-modified SSOs (e.g., 9/18_LNA_e58-1 (SSO1) and 9/18_AmNA_e58-1 (SSO2) in Figure 3a, and 6/18_LNA_e58-1 (SSO4) and 6/18_AmNA_e58-1 (SSO5) in Figure 3b). In addition, the melting temperature of the single-strand SSOs, *T*_m-ssSSO_, tended to be lower for the AmNA-modified SSOs than for the LNA-modified SSOs (Supplementary Figure S1). On the other hand, we could not observe definite tendencies of difference in hyperchromic effects between LNA-modified SSOs and GuNA-modified SSO, but the *T*_m-ssSSO_ of the GuNA-modified SSO was lower than that of the corresponding LNA-modified SSO in most cases (Supplementary Figure S1).

Next, we performed cell-based assays using stable-e58 cells, which stably express a minigene encoding *DMD* exons 57–59 for the evaluation of *DMD* exon 58 skipping (Figure 2b) [5]. A series of SSOs were transfected into these cells using Lipofectamine 2000 reagent. The RT-qPCR results showed that the AmNA-modified SSOs had higher exon 58 skipping activities than the LNA-modified SSOs. The 18-mer AmNA-modified SSO, 6/18_AmNA_e58-1 (Figure 2b, SSO5), showed the highest exon 58 skipping activity among the LNA- and AmNA-modified SSOs used in this study. In contrast, the GuNA-modified SSOs, except the 18-mer SSO 9/18_GuNA_e58-1 (SSO3), showed low exon 58 skipping activity compared with that of the LNA- and AmNA-modified SSOs. In particular, the SSOs with low GuNA content (e.g., 4/13_GuNA_e58-1 (SSO18), 6/13_GuNA_e58-1 (SSO15), and 5/15_GuNA_e58-1 (SSO12)) showed almost no exon skipping activity. However, most interestingly, the 18-mer GuNA-modified SSO, 9/18_GuNA_e58-1 (SSO3), showed the highest exon 58 skipping activity among all the SSOs used in this analysis.

### 2.2. Confirmation of the General Design of BNA-Modified SSOs Targeting DMD Exon 50 Using a Stably Transfected Cell Line

**Figure 4:**
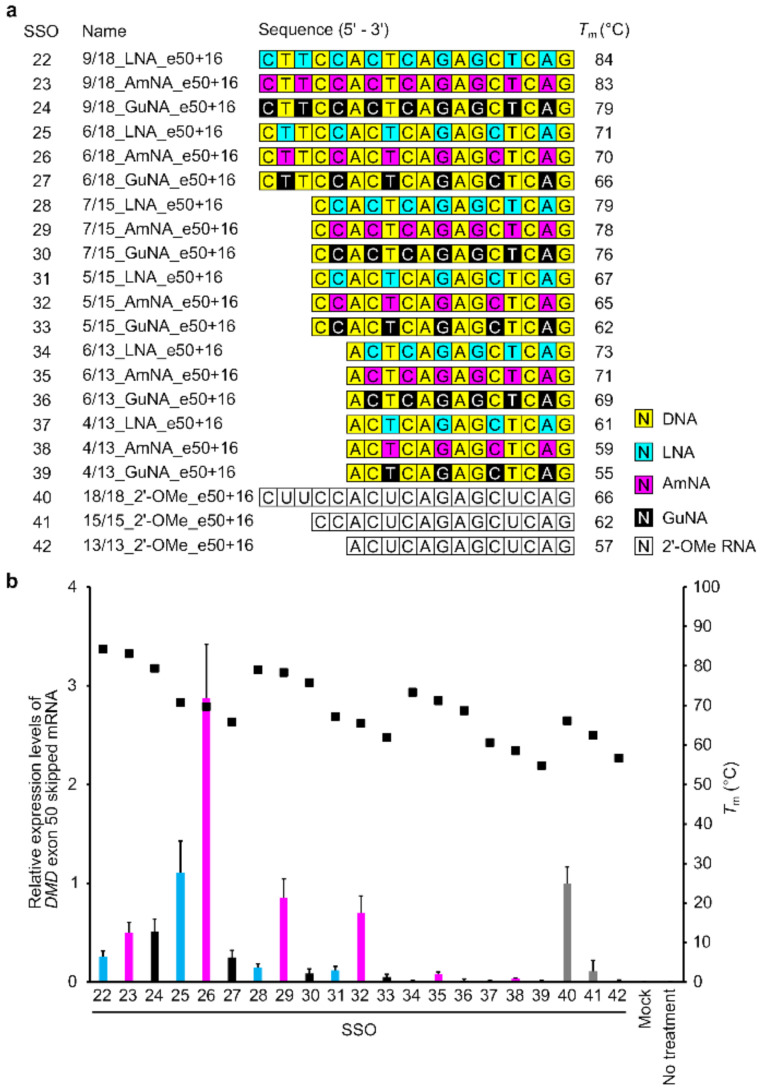
Design and evaluation of 13- to 18-mer LNA/AmNA/GuNA/2′-OMe RNA-modified SSOs targeting *DMD* exon 50 and having different lengths and modification content using stably transfected cells. (**a**) Schematic representation of 13- to 18-mer LNA, AmNA-, and GuNA, and 2′-OMe RNA-modified SSOs designed for the assay. (**b**) RT-qPCR analysis results. Stable-e50 cells were transfected with the indicated SSOs (10 nM) for 24 h. On the *x* axis of the graph, transfected SSOs are shown with SSO numbers mentioned in (**a**). The expression of exon 50-skipped mRNA was measured by RT-qPCR (bar). The expression was normalized to the *RPLP2* mRNA signal, relative to the value of 18/18_2′-OMe_e50+16 (SSO40) set to 1. Values represent the mean ± standard deviation of three independent experiments performed in duplicate. *T*_m_ value of SSOs was measured from 5 to 94.5 °C (black box). Mock: treated with Lipofectamine only; no treatment: no transfection. Values represent the mean ± standard deviation of three independent experiments.

To confirm the generality of the designs for AmNA- and GuNA-modified SSOs, we also synthesized SSOs targeting *DMD* exon 50 with different lengths (13-, 15-, and 18-mers) and different contents of LNA, AmNA, and GuNA (Figure 4a and Supplementary Table S3). To design SSOs targeting *DMD* exon 50, we first conducted a target site survey with a number of LNA-modified SSOs, referring to previous reports (Supplementary Figure S2) [24,25]. We then designed AmNA-modified and GuNA-modified SSOs with the same modification format as the LNA-modified SSOs. As in the case of SSOs for exon 58, the AmNA-modified SSOs showed similar or slightly lower *T*_m_ values than the corresponding LNA-modified SSOs, while the GuNA-modified SSOs showed *T_m_* values 3–6 °C lower than those of the LNA-modified SSOs (Figure 4a).

To evaluate the designed SSOs, we established a stable cell line containing a minigene expressing *DMD* exons 49–51 (stable-e50) according to our previous study (Supplementary Figure S3) [5,21]. A series of BNA-modified SSOs were transfected into these cells using Lipofectamine 2000. The results of RT-qPCR analysis revealed that both the 15-mer and 18-mer series of BNA-modified SSOs targeting *DMD* exon 50 possessed exon-skipping activities (Figure 4b). The AmNA-modified SSOs showed higher exon 50 skipping activities than the LNA-modified SSOs. In particular, the 18-mer AmNA-modified SSO (SSO 26; 6/18_AmNA_e50+16) showed the highest exon 50 skipping efficiency among all the assayed SSOs. In contrast, the GuNA-modified SSOs showed lower exon skipping activities than the corresponding LNA- and AmNA-modified SSOs, even with 18-mers. This is not consistent with the results observed with the SSOs targeting *DMD* exon 58.

**Figure 5:**
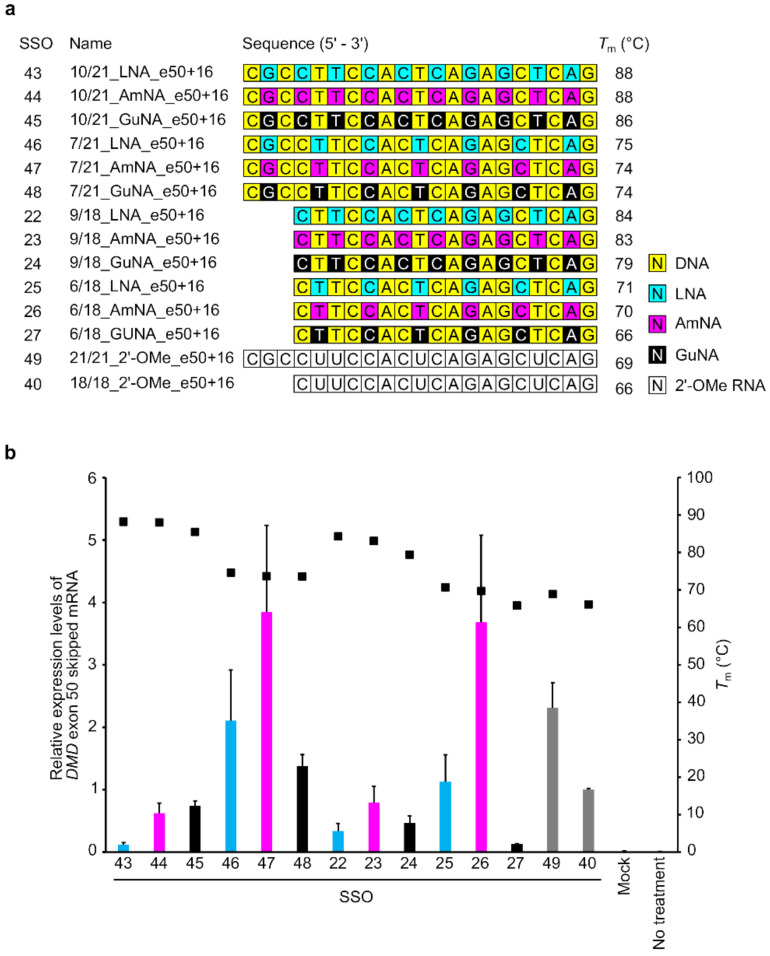
Design and evaluation of 21-mer LNA/AmNA/GuNA/2′-OMe RNA-modified SSOs targeting *DMD* exon 50 and having different lengths and modification content using stably transfected cells. (**a**) Schematic representation of 21-mer SSOs used for the assay. (**b**) RT-qPCR analysis results. Stable-e50 cells were transfected with the indicated SSOs (30 nM) for 24 h. On the *X*-axis of the graph, transfected SSOs are shown with SSO numbers mentioned in (**a**). The expression of exon 50-skipped mRNA was measured by RT-qPCR (bar). The expression was normalized to the *RPLP2* mRNA signal, relative to the value of 18/18_2′-OMe_e50+16 (SSO 40) set to 1. Values represent the mean ± standard deviation of three independent experiments performed in duplicate. *T*_m_ value of SSOs was measured from 5 to 94.5 °C (black box). Mock: treated with Lipofectamine only; no treatment: no transfection. Values represent the mean ± standard deviation of three independent experiments.

To further investigate the appropriate design of GuNA-modified SSOs, we prepared 21-mer SSOs for *DMD* exon 50 skipping (Figure 5a and Supplementary Table S5). The *T*_m_ values of these 21-mer SSOs against complementary RNA strands are shown in Figure 5a. Compared with the corresponding LNA-modified SSOs, the AmNA- and GuNA-modified SSOs showed slightly lower *T*_m_ values. Based on the in vitro activity evaluation using the stable-e50 cells, the 21-mer GuNA-modified SSOs 10/21_GuNA_e50+16 (Figure 5, SSO45) and 7/21_GuNA_e50+16 (SSO48) demonstrated increased activity compared with that of the corresponding 18-mer SSOs 9/18_GuNA_e50+16 (SSO24) and 6/18_GuNA_e50+16 (SSO27), whereas the 21-mer AmNA-modified SSOs 10/21_AmNA_e50+16 (SSO44) and 7/21_AmNA_e50+16 (SSO47) showed the same level of activity as the corresponding 18-mers 9/18_AmNA_e50+16 (SSO23) and 6/18_AmNA_e50+16 (SSO26). In the case of the LNA-modified SSOs, their activity was increased or decreased by increasing their sequence length to 21. In addition, the 21-mer SSOs 7/21_LNA_e50+16 (SSO46), 7/21_AmNA_e50+16 (SSO47), and 7/21_GuNA_e50+16 (SSO48), which have seven LNAs, AmNAs, and GuNAs, respectively, were more active than the corresponding SSOs with more modifications (10/21_LNA_e50+16 (SSO 43), 10/21_AmNA_e50+16 (SSO44), and 10/21_GuNA_e50+16 (SSO45)). Although the 21-mer SSOs with 10 modifications did not demonstrate high activity, it is interesting to note that when comparing the 10/21_LNA_e50+16 (SSO43), 10/21_AmNA_e50+16 (SSO44), and 10/21_GuNA_e50+16 (SSO45), the GuNA-modified SSO (SSO45) showed higher activity than the corresponding LNA-modified SSO (SSO43). Thus, in vitro analysis using two different stable cell lines (stable-e50 and stable-e58) revealed that the GuNA-modified SSOs tend to show higher exon skipping activities with longer lengths.

**Figure 6:**
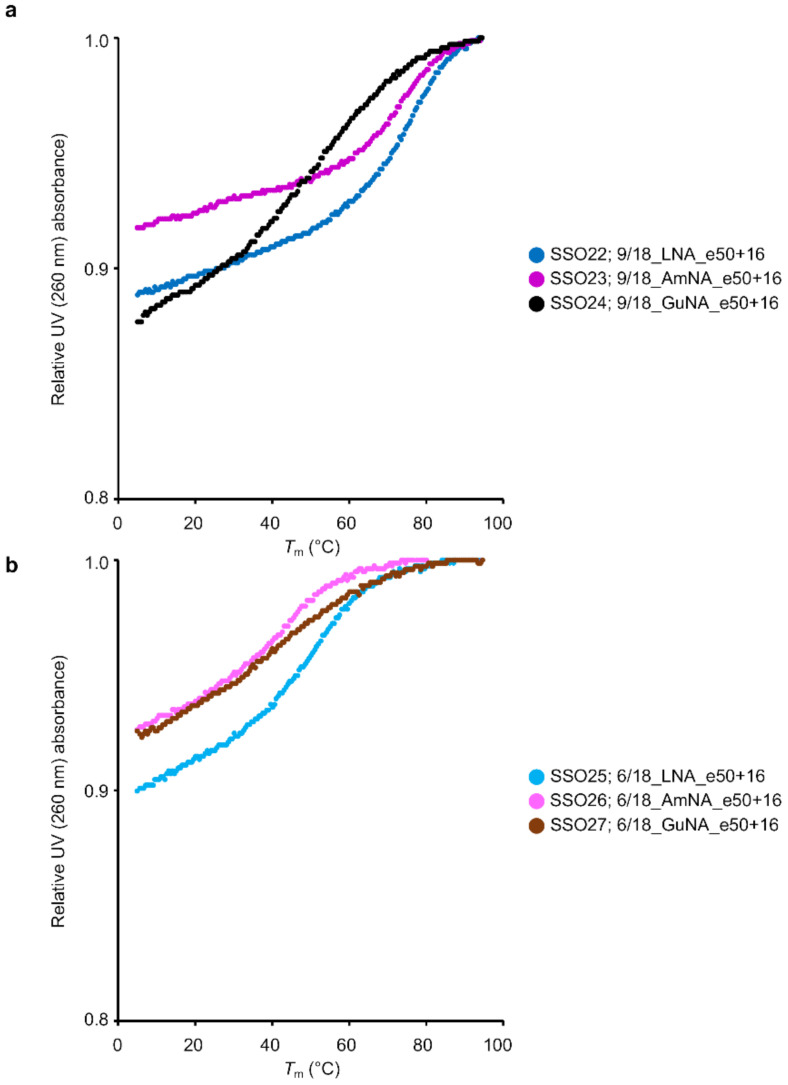
UV melting analysis of 18-mer LNA/AmNA/GuNA-modified SSOs targeting *DMD* exon 50 and having different modification content. (**a**) Results of UV melting experiments for single-stranded LNA-, AmNA-, and GuNA-modified SSOs having high modification content. (**b**) Results of UV melting experiments for single-stranded LNA-, AmNA-, and GuNA-modified SSOs having low modification content. All analyses were repeated three times to ensure reproducibility (see Supplementary Figure S5). The relative UV absorbance depicts normalized data (normalized by UV absorption at 94.5 °C set to 1).

We then performed UV melting analysis using the 18-mer and 21-mer SSOs without complementary oligonucleotides (Figure 6 and Supplementary Figures S4 and S5). The 18-mer AmNA-modified SSOs 9/18_AmNA_e50+16 (SSO24) and 6/18_AmNA_e50+16 (SSO25) showed lower hyperchromic effects than the corresponding 18-mer LNA-modified SSOs 9/18_LNA_e50+16 (SSO22) and 6/18_LNA_e50+16 (SSO25). The 21-mer AmNA-modified SSOs showed the same tendency. The GuNA-modified SSOs showed different hyperchromic effects depending on the content of the GuNA modification. In fact, the GuNA-modified SSO 9/18_GuNA_e50+16 (SSO24), despite having more GuNA modifications, showed almost the same hyperchromic effect as the LNA-modified SSOs 9/18_LNA_e50+16 (SSO22). However, the GuNA-modified SSO with fewer GuNA modifications (6/18_GuNA_e50+16 (SSO27)) showed a lower hyperchromic effect than the LNA-modified SSO 6/18_LNA_e50+16 (SSO25). We also confirmed that the 21-mer SSOs demonstrated the same tendency.

In addition, the *T*_m-ssSSO_ values of the AmNA-modified SSOs tended to be same or lower than the corresponding LNA-modified SSO values (Supplementary Figures S4 and S5). In contrast, the GuNA-modified SSOs showed varying absorptions and melting temperatures relative to the LNA-modified SSOs. Especially, 10/21_GuNA_e50+16 showed lower *T*_m-ssSSO_ than the corresponding LNA-modified SSO values (Supplementary Figures S4 and S5). Similar to the 18-mer SSOs, the 21-mer AmNA-modified SSOs showed a reduction in the hyperchromic effect compared with the LNA-modified SSOs (Supplementary Figure S5). This suggests that the AmNA modification prevents single-strand SSOs from forming secondary structures, although the detailed mechanism remains unknown.

### 2.3. Investigating the Effect on Dystrophin Protein Restoration of BNA-Modified SSOs Using DMD Model Cells

**Figure 7:**
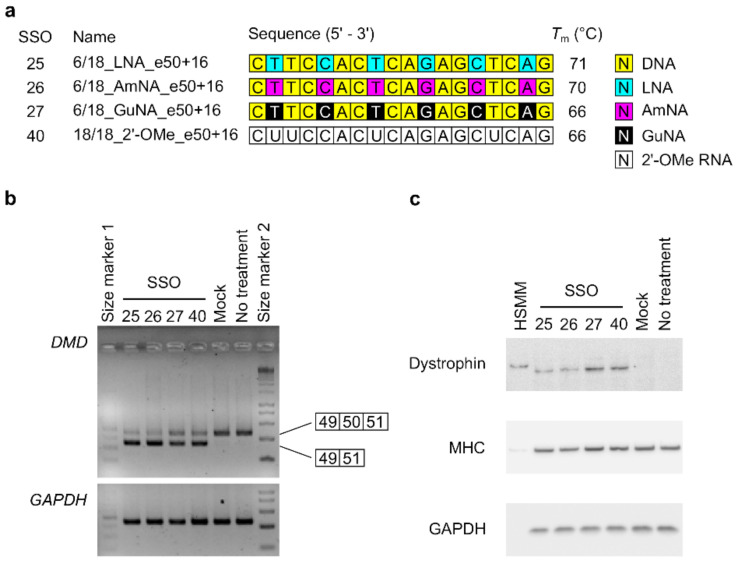
Evaluation of LNA/AmNA/GuNA-modified SSOs for *DMD* exon 50 skipping at both mRNA and protein levels in DMD model cells. (**a**) Schematic representation of 18-mer SSOs used for the assay. (**b**,**c**) Results of both RT-PCR and Western blotting analyses. Differentiated DMD model cells were transfected with the indicated SSOs (100 nM). (**b**) RT-PCR analysis shows the full-length (*DMD* exon 50 included) upper band (455 bp) and *DMD* exon 50 skipped lower band (346 bp). *GADPH* was used as an internal control. (**c**) Western blotting analysis. MHC: myosin heavy chain. Mock: treated with Lipofectamine only; no treatment: no transfection. The analysis was duplicated and repeated three times to ensure reproducibility.

Both the AmNA-modified and GuNA-modified SSOs induced *DMD* exon 58 and 50 skipping in the stable cells. Next, we sought to confirm whether these SSOs could restore dystrophin protein by inducing *DMD* exon 50 skipping in DMD model cells, human rhabdomyosarcoma cells with a *DMD* intron 50–57 deletion mutation induced by the CRISPR/Cas9 system [26]. We investigated the exon skipping activities of the SSOs in this DMD model cell line, according to our previous report [26]. In the present study, we used 18-mer SSOs containing six modifications for *DMD* exon 50 skipping (Figure 7a). RT-PCR analysis confirmed that both the AmNA-modified SSO (6/18_AmNA_e50+16 (SSO26)) and the GuNA-modified SSO (6/18_GuNA_e50+16 (SSO27)) possessed exon skipping activity (Figure 7b and Supplementary Figure S6). We calculated the ratio of exon 50 skipping (% exon 50 skipping) as the amount of exon 50 skipped transcript relative to the total transcript amount. Both the LNA-modified and AmNA-modified SSOs showed >60% exon 50 skipping. In addition, the GuNA-modified SSOs and 2′-OMe RNA SSOs showed ~50% exon 50 skipping. Additionally, Western blotting analysis showed that each SSO restored dystrophin protein levels (Figure 7c). Specifically, both the LNA- and AmNA-modified SSOs induced ~10% dystrophin restoration, and both the GuNA- and 2′-OMe RNA-modified SSOs induced ≥10% dystrophin protein restoration. Thus, this experiment succeeded in demonstrating that both the AmNA-modified SSOs and GuNA-modified SSOs can induce exon skipping at the mRNA level and restore dystrophin protein levels.

## 3. Discussion

In this study, we designed both AmNA- and GuNA-modified SSOs with different lengths (13-, 15-, 18-, and 21-mers) and modification content (approximately 33% and 50%). The in vitro study using stable-expressing cells revealed that both novel chemical modifications (AmNA and GuNA) show higher exon skipping activities than LNA modification when appropriately designed. In addition, both AmNA-modified and GuNA-modified SSOs restored dystrophin protein levels in the DMD model cell line [26].

We previously reported that LNA-modified SSOs for *DMD* exon 58 skipping require appropriate design, length, and LNA content in their sequence [5]. In the present study, we synthesized both AmNA- and GuNA-modified SSOs to reveal that each chemical modification requires optimization of the SSO design. As shown in Figure 4b and Figure 5b, the rules for designing AmNA-modified SSOs seem to be similar to those for LNA-modified SSOs; using 18-mer and 21-mer lengths and 33% modification content is suitable for both LNA- and AmNA-modified SSOs for *DMD* exon 50 skipping. Additionally, the 18-mer length and 33% modification content are appropriate for *DMD* exon 58 skipping (Figure 2b). In contrast, the design rules for GuNA-modified SSOs seem to be different from those of LNA-modified and AmNA-modified SSOs. In fact, the RT-qPCR analysis showed that a longer length enabled an increase in exon skipping efficiency, since the 21-mer GuNA-modified SSOs for *DMD* exon 50 and 18-mer GuNA-modified SSOs for *DMD* exon 58 showed higher exon skipping activities than the shorter GuNA-modified SSOs (Figure 2b and Figure 5b). The lower *T*_m_ of the GuNA-modified SSOs than that of the corresponding LNA-modified or AmNA-modified SSOs may explain the need to design a longer GuNA-modified SSO to obtain a high level of exon skipping activity. However, the difference in *T*_m_ between the GuNA-modified SSOs and LNA-modified or AmNA-modified SSOs is not as large as the sequence length-dependent difference in *T*_m_. Although it is compelling to presume that the binding affinity to the target RNA affects activity, the present results do not show a clear correlation between *T*_m_ and in vitro exon skipping activity. Overall, we can conclude that there are unique rules depending on the chemistry. Thus, screening using in vitro assays is important not only for target identification but also for designing the length and extent of modification.

In general, chemical modification is used to enhance splicing modulation by increasing binding affinity to the target RNA. It is also important to increase the stability of SSOs against nuclease activity. A recent study focused on additional specific characteristics, such as recruitment of splicing factors and inhibition of secondary structures. It was reported that a 2′-F RNA-modified SSO targeting *SMN2* intron 7 induced exon skipping, whereas the corresponding 2′-MOE-modified SSO promoted exon inclusion. This interesting result was explained by interaction of the 2′-F RNA-modified SSO with the interleukin enhancer binding factor 2 and 3 complex [14]. Although the interaction of SSOs with intracellular proteins requires further investigation, our present data do not support any such effect of either AmNA- or GuNA-modified SSOs, at least not the contrary effect seen with the 2′-F RNA-modified SSO. In 2019, we indicated that the incorporation of base modifications, such as 7-deaza-2-deoxyguanosine or 2′-deoxyinosine, into SSOs increases exon skipping activities by inhibiting secondary structure formation by the SSOs [6]. In the study, we observed that the 17-mer LNA-modified SSOs targeting *DMD* exon 58 formed secondary structures, and inhibition of these structures by introducing base modifications (7-deaza-2-deoxyguanosine or 2′-deoxyinosine) increased exon skipping efficiencies. The UV melting experiment indicated that the AmNA-modified SSOs had reduced secondary structures, although there has been no report to our knowledge about AmNA analogs inhibiting secondary structure formation.

AmNA-modified SSOs with short lengths showed better *DMD* exon 50 skipping efficiencies than either the LNA- or GuNA-modified SSOs; the 15-mer AmNA-modified SSOs (SSO 29; 7/15_AmNA_e50+16 and SSO 32; 5/15_AmNA_e50+16) induced exon skipping, while the 15-mer SSOs with other chemistries did not. The AmNA-modified SSOs for *DMD* exon 58 skipping showed similar results; the 13-mer AmNA-modified SSOs showed higher exon skipping efficiencies than either the 13-mer LNA- or GuNA-modified SSOs. This appears to be another characteristic of AmNA-modified SSOs. We suggest that the AmNA analog is suitable for the initial screening of SSOs for two reasons: (1) initial screening for target identification with short SSOs reduces costs because SSO screening requires the synthesis of many candidate SSOs, and (2) the higher exon skipping induction of AmNA-modified SSOs enables the detection of many more potential SSO target sites.

The results of this study could reveal that chemical modifications enhance exon skipping activities by increasing binding affinities against RNA as we and others previously reported [5,6,7,8,9,10,11,12,13]. On the other hand, Scharner et al. mentioned that the kinds of chemical modification vary the off-target effect of SSOs [27]. Thus, it is important to consider the off-target effects of SSOs, having chemical modifications as well in case of designing the SSOs. Our previous report revealed that the different length of SSOs change the off-target effects. Additionally, Pires et al. mentioned that the appropriate length of LNA-modified SSO enables the prevention of off-target effects [28]. Although further study is necessary to determine the off-target effects of BNA-modified SSOs, we at least suggest that both AmNA and GuNA are candidates of chemical modifications for better splicing modulation.

In conclusion, we have introduced a series of BNAs—namely, LNA, AmNA, and GuNA—into SSOs and evaluated their exon skipping efficiencies in vitro. As we summarized our findings in this study (Table 1), we found that both the AmNA and GuNA analogs lead to an increase in exon skipping when appropriately designed. We also found that the AmNA analogs might increase exon skipping by reducing the secondary structures in SSOs.

## 4. Materials and Methods

### 4.1. Synthesis of Oligonucleotides

All the SSOs and complementary RNA strands used in this study are shown in Supplementary Tables S1–S6 and S10. Chemical modifications—namely, LNA, AmNA, GuNA, and 2′-OMe RNA—were used for the SSO sequences, in which the phosphodiester linkages were completely replaced with phosphorothioate (PS) linkages. LNA- or 2′-OMe RNA-modified SSOs designed to have sequences complementary to the human *DMD* gene were synthesized and purified by GeneDesign Inc. (Osaka, Japan). AmNA- or GuNA-modified SSOs were also synthesized and purified by GeneDesign Inc. (Osaka, Japan) by using the corresponding AmNA- or GuNA-phosphoramidites [16,17,29,30]. We gave simple names to each SSO, e.g., 6/18_LNA_e58-1. This name reflects the number of BNA-modifications, the length of SSOs, kinds of BNA-modifications, target exon, and the target site of SSOs (5′-end of targeted exon).

All DNA primers used in this study are shown in Supplementary Tables S7–S9. The primers were designed to be complementary to the human *DMD* gene, human *ribosomal protein lateral stalk subunit P2* (*RPLP2*) gene, and human *glyceraldehyde-3-phosphate dehydrogenase* (*GAPDH*) gene, and were synthesized and purified by Hokkaido System Sciences Inc. (Hokkaido, Japan).

### 4.2. UV Melting Analysis

The melting temperature (*T*_m_) of each SSO against its complementary RNA strand was measured as reported in our previous study [5]. In brief, each SSO and native complementary RNA oligonucleotide were dissolved in 10 mM sodium phosphate buffer (pH 7.2) containing 10 mM NaCl to a final concentration of 2 µM. The absorbance at 260 nm was measured from 5 °C to 94.5 °C at a scan rate of 0.5 °C/min. The peak temperature in the derivative curve was the *T*_m_ value.

To investigate the secondary structures formed by single-stranded SSOs, each SSO was dissolved in 10 mM sodium phosphate buffer (pH 7.2) containing 100 mM NaCl to a final concentration of 4 µM. The absorbance at 260 nm was measured from 5 °C to 94.5 °C at a scan rate of 0.5 °C/min.

### 4.3. Cell Culture

Both stably transfected cells, stable-e58 [5] and stable-e50, were cultured in high-glucose Dulbecco’s modified Eagle medium (DMEM) containing 10% fetal bovine serum (FBS) (Biowest, Nuaillé, France), 1× antibiotic-antimycotic (A.A.) solution for cell culture (Sigma-Aldrich, St. Louis, MO, USA), and 100 µg/mL hygromycin B (Thermo Fisher Scientific, Waltham, MA, USA) and were maintained in a 5% CO_2_ incubator at 37 °C. The DMD model cells [26] were cultured in high-glucose DMEM containing 10% FBS (Biowest) and 1× A.A. solution for cell culture (Sigma-Aldrich) and were maintained in a 5% CO_2_ incubator at 37 °C.

### 4.4. SSO Transfection

For transfection experiments, we used three cell lines: two stable-expressing cells, stable-e58 and stable-e50, and the DMD model cells. The stable cells were seeded 1 day before SSO transfection at a density of 200,000 cells/well in 24-well plates (Iwaki Techno Glass, Tokyo, Japan). After 24 h, the cells were transfected with SSOs at a concentration of 10 nM (Figure 2b and Figure 4b) or 30 nM (Figure 5b) using Lipofectamine 2000 according to the manufacturer’s protocols and then grown in high-glucose DMEM containing 10% FBS and 1× A.A solution. Twenty-four hours after SSO transfection, the cells were harvested and used for assays.

The DMD model cells were seeded 7 days before SSO transfection at a density of 400,000 cells/well in collagen type 1-coated 12-well plates (Iwaki Techno Glass). One day after cell seeding, the medium was changed to differentiation medium, which contained 100 nM 12-*O*-Tetradecanoylphorbol-13-Acetate (TPA). The cells were transfected with SSOs at a concentration of 100 nM (Figure 7) or 500 nM (Supplementary Figure S2), each using Lipofectamine 2000 according to the manufacturer’s protocols. Twenty-four hours (RT-PCR) or 96 h (Western blotting) after SSO transfection, the cells were harvested and used for assays.

### 4.5. RNA Isolation and cDNA Synthesis

Twenty-four hours after SSO transfection, total RNA was isolated from the samples using a QuickGene-800 apparatus (Kurabo, Osaka, Japan), QuickGene RNA Cultured Cell Kit S (Kurabo), and RQ1 RNase-Free DNase (Promega, Madison, WI, USA) according to the manufacturer’s instructions. Total RNA was reverse transcribed using the ReverTra Ace qPCR RT Master Mix (Toyobo, Osaka, Japan) according to the manufacturer’s instructions.

### 4.6. RT-PCR Analysis

RT-PCR analysis were performed according to our previous reports [5,26,31]. We used specific primer sets (Supplementary Table S7). The housekeeping gene, *GAPDH,* was used as an internal control.

### 4.7. Quantitative RT-PCR Analysis

cDNA (0.1 ng/µL) was used as the template for individual PCRs using specific primer sets (Supplementary Tables S8 and S9), which were designed using the Primer-BLAST program [32]. Quantitative RT-PCR (RT-qPCR) analyses were performed as described in our previous study [26]. The expression of human *RPLP2* mRNA was used to normalize the data. The amplification specificity of the PCR products was assessed on a 5% agarose gel stained with ethidium bromide and via melting curve analysis of the qPCR products.

### 4.8. Immunoblot Analysis

Western blot analyses were performed as previously reported [26]. Total protein extracted from differentiated human skeletal muscle myoblasts (HSMMs, Lonza, Walkersville, MD, USA) was used as a positive control of dystrophin protein expression (427 kDa). The HSMMs were cultured in T-75 flasks in DMEM containing 10% FBS and antibiotics. When the cells reached >80% confluence, they were differentiated by changing the medium to DMEM containing 2% horse serum (Thermo Fisher Scientific) with antibiotics for 10 days.

## Figures and Tables

**Table 1:** Summary of BNA-modified SSOs in this study.

Type of BNA	Target Exon	Minimal Length for Effective Exon Skipping	*T*_m_ (°C) for Effective Exon Skipping	Key Features
LNA	DMD exon 58	13	72 to 85	–
	DMD exon 50	18	71 to 75	
AmNA	DMD exon 58	13	71 to 85	Higher activity than the corresponding LNA-modified SSOs
	DMD exon 50	15	65 to 78	
GuNA	DMD exon 58	15	79 to 82	Longer SSO is more active
	DMD exon 50	21	74	

## Data Availability

Not applicable.

## Supplementary Materials

The following are available online at https://www.mdpi.com/article/10.3390/ijms22073526/s1, Supplementary Materials and Methods, Figure S1: UV melting analysis for 18-mer LNA/AmNA/GuNA-modified SSOs targeting *DMD* exon 58 having different modification content, Figure S2: Screening of 15-mer LNA-modified SSOs targeting *DMD* exon 50 using the DMD model cell line, Figure S3: Schematic representation of the DMD minigene and its splicing pattern, Figure S4: UV melting analysis for 18-mer LNA/AmNA/GuNA-modified SSOs targeting *DMD* exon 50 having different modification content, Figure S5: UV melting analysis for 21-mer LNA/AmNA/GuNA-modified SSOs targeting *DMD* exon 50 having different modification content, Figure S6: Evaluation of LNA/AmNA/GuNA-modified SSOs for *DMD* exon 50 skipping at mRNA levels in DMD model cells, Table S1: LNA/AmNA/GuNA/2′-OMe RNA-based SSOs targeting *DMD* exon 58 used for the experiment, Table S2: Complementary RNA used for UV melting analysis of SSOs targeting *DMD* exon 58, Table S3: LNA/AmNA/GuNA/2′-OMe RNA-based SSOs targeting *DMD* exon 50 used for the experiment, Table S4: Complementary RNA used for UV melting analysis of SSOs targeting *DMD* exon 50, Table S5: 21-mer BNA-based SSOs targeting *DMD* exon 50 used for the experiment, Table S6: Complementary RNA used for UV melting analysis of 21-mer SSOs targeting *DMD* exon 50, Table S7: Primers used for RT-PCR analysis investigating *DMD* exon 50 skipping, Table S8: Primers used for quantitative PCR analysis investigating *DMD* exon 58 skipping, Table S9: Primers used for quantitative PCR analysis investigating *DMD* exon 50 skipping, Table S10: LNA-based SSOs used for target selection of *DMD* exon 50.

## References

[1] Hwang J., Yokota T. (2019). Recent advancements in exon-skipping therapies using antisense oligonucleotides and genome editing for the treatment of various muscular dystrophies. Expert Rev. Mol. Med..

[2] Havens M.A., Hastings M.L. (2016). Splice-switching antisense oligonucleotides as therapeutic drugs. Nucleic Acids Res..

[3] Roberts T.C., Langer R., Wood M.J.A. (2020). Advances in oligonucleotide drug delivery. Nat. Rev. Drug Discov..

[4] Sheng L., Rigo F., Bennett C.F., Krainer A.R., Hua Y. (2020). Comparison of the efficacy of MOE and PMO modifications of systemic antisense oligonucleotides in a severe SMA mouse model. Nucleic Acids Res..

[5] Shimo T., Tachibana K., Saito K., Yoshida T., Tomita E., Waki R., Yamamoto T., Doi T., Inoue T., Kawakami J. (2014). Design and evaluation of locked nucleic acid-based splice-switching oligonucleotides in vitro. Nucleic Acids Res..

[6] Shimo T., Tachibana K., Kawawaki Y., Watahiki Y., Ishigaki T., Nakatsuji Y., Hara T., Kawakami J., Obika S. (2019). Enhancement of exon skipping activity by reduction in the secondary structure content of LNA-based splice-switching oligonucleotides. Chem. Commun..

[7] Le B.T., Murayama K., Shabanpoor F., Asanuma H., Veedu R.N. (2017). Antisense oligonucleotide modified with serinol nucleic acid (SNA) induces exon skipping in mdx myotubes. RSC Adv..

[8] Le B.T., Adams A.M., Fletcher S., Wilton S.D., Veedu R.N. (2017). Rational Design of Short Locked Nucleic Acid-Modified 2’-O-Methyl Antisense Oligonucleotides for Efficient Exon-Skipping In Vitro. Mol. Ther. Nucleic Acids.

[9] Chen S., Le B.T., Chakravarthy M., Kosbar T.R., Veedu R.N. (2019). Systematic evaluation of 2’-Fluoro modified chimeric antisense oligonucleotide-mediated exon skipping in vitro. Sci. Rep..

[10] Le B.T., Chen S., Abramov M., Herdewijn P., Veedu R.N. (2016). Evaluation of anhydrohexitol nucleic acid, cyclohexenyl nucleic acid and d-altritol nucleic acid-modified 2’-O-methyl RNA mixmer antisense oligonucleotides for exon skipping in vitro. Chem. Commun..

[11] Roberts J., Palma E., Sazani P., Orum H., Cho M., Kole R. (2006). Efficient and persistent splice switching by systemically delivered LNA oligonucleotides in mice. Mol. Ther..

[12] Surono A., Van Khanh T., Takeshima Y., Wada H., Yagi M., Takagi M., Koizumi M., Matsuo M. (2004). Chimeric RNA/ethylene-bridged nucleic acids promote dystrophin expression in myocytes of duchenne muscular dystrophy by inducing skipping of the nonsense mutation-encoding exon. Hum. Gene Ther..

[13] Jarver P., O’Donovan L., Gait M.J. (2014). A chemical view of oligonucleotides for exon skipping and related drug applications. Nucleic Acid Ther..

[14] Rigo F., Hua Y., Chun S.J., Prakash T.P., Krainer A.R., Bennett C.F. (2012). Synthetic oligonucleotides recruit ILF2/3 to RNA transcripts to modulate splicing. Nat. Chem. Biol..

[15] Kawasaki A.M., Casper M.D., Freier S.M., Lesnik E.A., Zounes M.C., Cummins L.L., Gonzalez C., Cook P.D. (1993). Uniformly modified 2’-deoxy-2’-fluoro phosphorothioate oligonucleotides as nuclease-resistant antisense compounds with high affinity and specificity for RNA targets. J. Med. Chem..

[16] Yahara A., Shrestha A.R., Yamamoto T., Hari Y., Osawa T., Yamaguchi M., Nishida M., Kodama T., Obika S. (2012). Amido-bridged nucleic acids (AmNAs): Synthesis, duplex stability, nuclease resistance, and in vitro antisense potency. Chembiochem.

[17] Shrestha A.R., Kotobuki Y., Hari Y., Obika S. (2014). Guanidine bridged nucleic acid (GuNA): An effect of a cationic bridged nucleic acid on DNA binding affinity. Chem. Commun..

[18] Popplewell L.J., Trollet C., Dickson G., Graham I.R. (2009). Design of phosphorodiamidate morpholino oligomers (PMOs) for the induction of exon skipping of the human DMD gene. Mol. Ther..

[19] Disterer P., Kryczka A., Liu Y., Badi Y.E., Wong J.J., Owen J.S., Khoo B. (2014). Development of therapeutic splice-switching oligonucleotides. Hum. Gene. Ther..

[20] Popplewell L.J., Malerba A., Dickson G. (2012). Optimizing antisense oligonucleotides using phosphorodiamidate morpholino oligomers. Methods Mol. Biol..

[21] Shimo T., Obika S. (2018). Optimization of 2’,4’-BNA/LNA-Based Oligonucleotides for Splicing Modulation In Vitro. Methods Mol. Biol..

[22] Graziewicz M.A., Tarrant T.K., Buckley B., Roberts J., Fulton L., Hansen H., Orum H., Kole R., Sazani P. (2008). An Endogenous TNF-alpha Antagonist Induced by Splice-switching Oligonucleotides Reduces Inflammation in Hepatitis and Arthritis Mouse Models. Mol. Ther..

[23] Bestas B., Moreno P.M., Blomberg K.E., Mohammad D.K., Saleh A.F., Sutlu T., Nordin J.Z., Guterstam P., Gustafsson M.O., Kharazi S. (2014). Splice-correcting oligonucleotides restore BTK function in X-linked agammaglobulinemia model. J. Clin. Investig..

[24] Aartsma-Rus A., De Winter C.L., Janson A.A., Kaman W.E., Van Ommen G.J., Den Dunnen J.T., Van Deutekom J.C. (2005). Functional analysis of 114 exon-internal AONs for targeted DMD exon skipping: Indication for steric hindrance of SR protein binding sites. Oligonucleotides.

[25] Wu B., Lu P., Cloer C., Shaban M., Grewal S., Milazi S., Shah S.N., Moulton H.M., Lu Q.L. (2012). Long-term rescue of dystrophin expression and improvement in muscle pathology and function in dystrophic mdx mice by peptide-conjugated morpholino. Am. J. Pathol..

[26] Shimo T., Hosoki K., Nakatsuji Y., Yokota T., Obika S. (2018). A novel human muscle cell model of Duchenne muscular dystrophy created by CRISPR/Cas9 and evaluation of antisense-mediated exon skipping. J. Hum. Genet..

[27] Scharner J., Ma W.K., Zhang Q., Lin K.T., Rigo F., Bennett C.F., Krainer A.R. (2020). Hybridization-mediated off-target effects of splice-switching antisense oligonucleotides. Nucleic Acids Res..

[28] Pires V.B., Simoes R., Mamchaoui K., Carvalho C., Carmo-Fonseca M. (2017). Short (16-mer) locked nucleic acid splice-switching oligonucleotides restore dystrophin production in Duchenne Muscular Dystrophy myotubes. PLoS ONE.

[29] Yamamoto T., Yahara A., Waki R., Yasuhara H., Wada F., Harada-Shiba M., Obika S. (2015). Amido-bridged nucleic acids with small hydrophobic residues enhance hepatic tropism of antisense oligonucleotides in vivo. Org. Biomol. Chem..

[30] Kumagai S., Sawamoto H., Takegawa-Araki T., Arai Y., Yamakoshi S., Yamada K., Ohta T., Kawanishi E., Horie N., Yamaguchi T. (2020). Synthesis and properties of GuNA purine/pyrimidine nucleosides and oligonucleotides. Org. Biomol. Chem..

[31] Shimo T., Tachibana K., Obika S. (2018). Construction of a tri-chromatic reporter cell line for the rapid and simple screening of splice-switching oligonucleotides targeting DMD exon 51 using high content screening. PLoS ONE.

[32] Ye J., Coulouris G., Zaretskaya I., Cutcutache I., Rozen S., Madden T.L. (2012). Primer-BLAST: A tool to design target-specific primers for polymerase chain reaction. BMC Bioinform..

