# Supplementary material for: Design and In Vitro Evaluation of Splice-Switching Oligonucleotides Bearing Locked Nucleic Acids, Amido-Bridged Nucleic Acids, and Guanidine-Bridged Nucleic Acids
Source: Int J Mol Sci. 2021 Mar 29;22(7):3526. doi: 10.3390/ijms22073526 (PMC8037388; doi:10.3390/ijms22073526)
Supplement: Supplementary file 1 [file ijms-22-03526-s001.pdf]

Supplementary Information

Design and In Vitro Evaluation of Splice-Switching Oligonucleotides Bearing Locked Nucleic Acids, Amido-Bridged Nucleic Acids, and Guanidine-Bridged Nucleic Acids

Takenori Shimo, Yusuke Nakatsuji, Keisuke Tachibana and Satoshi Obika *

Graduate School of Pharmaceutical Sciences, Osaka University, 1-6 Yamadaoka, Suita, Osaka 565-0871, Japan; shimo-t@phs.osaka-u.ac.jp (T.S.); nktj101@outlook.jp (Y.N.); nya@phs.osaka-u.ac.jp (K.T.)

* Correspondence: obika@phs.osaka-u.ac.jp; Tel.: +81-6-6879-8200

SUPPLEMENTARY MATERIALS AND METHODS

Experimental details

Optimization of LNA-modified SSOs targeting *DMD* exon 50

To optimize the target sequence, we used LNA-based SSOs, which contained seven LNA analogs in 15-mer DNA-based oligonucleotides according to our previous report.[5] We also referred to two previous reports focusing on *DMD* exon 50 skipping by 2′-OMe RNA- and PMO-based SSOs.[24, 25] We synthesized five LNA-based SSOs, targeting +4+18, +8+22, +12+26, +16+30, and +20+34 on *DMD* exon 50 to explore the possible target sites for effective *DMD* exon 50 skipping (Supplementary Figures S1A and S1B and Table S10). We also synthesized seven SSOs targeting +83+97, +87+101, +91+105, +95+109, +99-4, +103-8, and +107-12 to explore the possible target sites for effective *DMD* exon 50 skipping (Supplementary Figures S1A and S1B and Table S10). RT-PCR analysis using DMD model cells revealed that three LNA-based SSOs, +16+30, +20+34, and +83+97, showed higher exon skipping efficiencies than the other LNA-based SSOs used in this study (Supplementary Figure S1C). The results also showed that the exon skipping activities of three LNA-based SSOs, +16+30, +20+34, and +83+97, showed higher exon skipping activities than h50AON1, which is a 2′-

OMe RNA-based SSO named by Aartsma-Rus *et al.* In summary, we identified appropriate target sites for comparing the exon skipping efficiencies of LNA-, AmNA-, and GuNA-based SSOs.

Plasmid construction

The DNA fragments that encode *DMD* exons 49, 50, and 51, including the shortened introns 49 and 50, were synthesized using the gBlocks gene fragment service from Integrated DNA Technologies (Coralville, IA, USA). Both the synthesized DNA fragment and plasmid DNA (pcDNA5/FRT-Flag-NLS-DMD-exon50-51-52-EGFP-TagRFP)[31] were digested with the restriction enzymes AgeI-HF and BstBI. The DNA fragment were then inserted into the plasmid DNA using the In-Fusion HD Cloning Kit (pcDNA5/FRT-Flag-NLS-DMD-exon49-50-51-EGFP-TagRFP). We used this plasmid DNA as the minigene for *DMD* exon 50 skipping. The construct was verified by DNA sequencing.

Establishment of stable cell line

The Flp-In 293 cell line was used to establish a stable cell line. Flp-In 293

cells were seeded in a 6-well plate (Iwaki Techno Glass, Tokyo, Japan) 24 h before transfection. The plasmid DNAs (0.6 ng/well pcDNA5/FRT-Flag-NLS-DMD-exon49-50-51-EGFP-TagRFP and 5.4 ng/well pOG44 (the Flp recombinase expression plasmid) were co-transfected into the Flp-In 293 cells using 12.5 μL/well of Lipofectamine 2000 in 2.5 mL of culture medium. Six hours after transfection, the medium was changed. Two days after transfection, stable cells were selected using 50 μg/mL hygromycin B and called Flp-In 293-DMD-exon50-GFP-RFP. The stable cells were cultured in DMEM containing 10% FBS, 1× antibiotic-antimycotic solution, and 100 μg/mL hygromycin B.

Supplementary Table S1. LNA/AmNA/GuNA/2′-OMe RNA-based SSOs targeting *DMD* exon 58 used for the experiment. Twenty-one SSOs for *DMD* exon 58 skipping are shown. Sequences are shown from 5′ to 3′. Lowercase letter: DNA, uppercase character or 5 with (L): LNA, uppercase character or 5 with (A): AmNA, uppercase character or 5 with (G): GuNA, uppercase character with (M): 2′-OMe RNA, 5: 5-methylcytosine, and ^: phosphorothioate.

SSO	Name	Sequence	T_m value (°C)
1	9/18_LNA_e58-1	A(L)^a^T(L)^t^5(L)^c^5(L)^t^5(L)^t^T(L)^g^A(L)^a^G(L)^g^5(L)^c	85
2	9/18_AmNA_e58-1	A(A)^a^T(A)^t^5(A)^c^5(A)^t^5(A)^t^T(A)^g^A(A)^a^G(A)^g^5(A)^c	85
3	9/18_GuNA_e58-1	A(G)^a^T(G)^t^5(G)^c^5(G)^t^5(G)^t^T(G)^g^A(G)^a^G(G)^g^5(G)^c	82
4	6/18_LNA_e58-1	a^A(L)^t^t^5(L)^c^c^T(L)^c^t^T(L)^g^a^A(L)^g^g^5(L)^c	72
5	6/18_AmNA_e58-1	a^A(A)^t^t^5(A)^c^c^T(A)^c^t^T(A)^g^a^A(A)^g^g^5(A)^c	71
6	6/18_GuNA_e58-1	a^A(G)^t^t^5(G)^c^c^T(G)^c^t^T(G)^g^a^A(G)^g^g^5(G)^c	70
7	7/15_LNA_e58-1	t^5(L)^c^5(L)^t^5(L)^t^T(L)^g^A(L)^a^G(L)^g^5(L)^c	83
8	7/15_AmNA_e58-1	t^5(A)^c^5(A)^t^5(A)^t^T(A)^g^A(A)^a^G(A)^g^5(A)^c	82
9	7/15_GuNA_e58-1	t^5(G)^c^5(G)^t^5(G)^t^T(G)^g^A(G)^a^G(G)^g^5(G)^c	79
10	5/15_LNA_e58-1	t^5(L)^c^c^T(L)^c^t^T(L)^g^a^A(L)^g^g^5(L)^c	71
11	5/15_AmNA_e58-1	t^5(A)^c^c^T(A)^c^t^T(A)^g^a^A(A)^g^g^5(A)^c	71
12	5/15_GuNA_e58-1	t^5(G)^c^c^T(G)^c^t^T(G)^g^a^A(G)^g^g^5(G)^c	69
13	6/13_LNA_e58-1	c^5(L)^t^5(L)^t^T(L)^g^A(L)^a^G(L)^g^5(L)^c	75

14	6/13_AmNA_e58-1	c^5(A)^t^5(A)^t^T(A)^g^A(A)^a^G(A)^g^5(A)^c	74
15	6/13_GuNA_e58-1	c^5(G)^t^5(G)^t^T(G)^g^A(G)^a^G(G)^g^5(G)^c	71
16	4/13_LNA_e58-1	c^c^T(L)^c^t^T(L)^g^a^A(L)^g^g^5(L)^c	60
17	4/13_AmNA_e58-1	c^c^T(A)^c^t^T(A)^g^a^A(A)^g^g^5(A)^c	60
18	4/13_GuNA_e58-1	c^c^T(G)^c^t^T(G)^g^a^A(G)^g^g^5(G)^c	58
19	18/18_2′-OMe _e58-1	A(M)^A(M)^U(M)^U(M)^C(M)^C(M)^C(M)^U(M)^C(M)^U(M)^U(M)^G(M)^A(M)^A (M)^G(M)^G(M)^C(M)^C(M)	66
20	15/15_2′-OMe _e58-1	U(M)^C(M)^C(M)^C(M)^U(M)^C(M)^U(M)^U(M)^G(M)^A(M)^A(M)^G(M)^G(M)^C (M)^C(M)	65
21	13/13_2′-OMe _e58-1	C(M)^C(M)^U(M)^C(M)^U(M)^U(M)^G(M)^A(M)^A(M)^G(M)^G(M)^C(M)^C(M)	59

Supplementary Table S2. Complementary RNA used for UV melting analysis of SSOs targeting *DMD* exon 58. Sequences are shown from 5′ to 3′.

Entry	Name	Sequence
C-1	Comp.RNA_18/18_RNA_e58-1	GGCCUUCAAGAGGGAAUU
C-2	Comp.RNA_15/15_RNA_e58-1	GGCCUUCAAGAGGGA
C-3	Comp.RNA_13/13_RNA_e58-1	GGCCUUCAAGAGG

Supplementary Table S3. LNA/AmNA/GuNA/2′-OMe RNA-based SSOs targeting *DMD* exon 50 used for the experiment. Twenty-one SSOs for *DMD* exon 50 skipping are shown. Sequences are shown from 5′ to 3′. Lowercase letter: DNA, uppercase character or 5 with (L): LNA, uppercase character or 5 with (A): AmNA, uppercase character or 5 with (G): GuNA, uppercase character with (M): 2′-OMe RNA, 5: 5-methylcytosine, and ^: phosphorothioate.

SSO	Name	Sequence	T_m value (°C)
22	9/18_LNA_e50+16	5(L)^t^T(L)^c^5(L)^a^5(L)^t^5(L)^a^G(L)^a^G(L)^c^T(L)^c^A(L)^g	84
23	9/18_AmNA_e50+16	5(A)^t^T(A)^c^5(A)^a^5(A)^t^5(A)^a^G(A)^a^G(A)^c^T(A)^c^A(A)^g	83
24	9/18_GuNA_e50+16	5(G)^t^T(G)^c^5(G)^a^5(G)^t^5(G)^a^G(G)^a^G(G)^c^T(G)^c^A(G)^g	79
25	6/18_LNA_e50+16	c^T(L)^t^c^5(L)^a^c^T(L)^c^a^G(L)^a^g^5(L)^t^c^A(L)^g	71
26	6/18_AmNA_e50+16	c^T(A)^t^c^5(A)^a^c^T(A)^c^a^G(A)^a^g^5(A)^t^c^A(A)^g	70
27	6/18_GuNA_e50+16	c^T(G)^t^c^5(G)^a^c^T(G)^c^a^G(G)^a^g^5(G)^t^c^A(G)^g	66
28	7/15_LNA_e50+16	c^5(L)^a^5(L)^t^5(L)^a^G(L)^a^G(L)^c^T(L)^c^A(L)^g	79
29	7/15_AmNA_e50+16	c^5(A)^a^5(A)^t^5(A)^a^G(A)^a^G(A)^c^T(A)^c^A(A)^g	78

30	7/15_GuNA_e50+16	c^5(G)^a^5(G)^t^5(G)^a^G(G)^a^G(G)^c^T(G)^c^A(G)^g	76
31	5/15_LNA_e50+16	c^5(L)^a^c^T(L)^c^a^G(L)^a^g^5(L)^t^c^A(L)^g	67
32	5/15_AmNA_e50+16	c^5(A)^a^c^T(A)^c^a^G(A)^a^g^5(A)^t^c^A(A)^g	65
33	5/15_GuNA_e50+16	c^5(G)^a^c^T(G)^c^a^G(G)^a^g^5(G)^t^c^A(G)^g	62
34	6/13_LNA_e50+16	a^5(L)^t^5(L)^a^G(L)^a^G(L)^c^T(L)^c^A(L)^g	73
35	6/13_AmNA_e50+16	a^5(A)^t^5(A)^a^G(A)^a^G(A)^c^T(A)^c^A(A)^g	71
36	6/13_GuNA_e50+16	a^5(G)^t^5(G)^a^G(G)^a^G(G)^c^T(G)^c^A(G)^g	69
37	4/13_LNA_e50+16	a^c^T(L)^c^a^G(L)^a^g^5(L)^t^c^A(L)^g	61
38	4/13_AmNA_e50+16	a^c^T(A)^c^a^G(A)^a^g^5(A)^t^c^A(A)^g	59
39	4/13_GuNA_e50+16	a^c^T(G)^c^a^G(G)^a^g^5(G)^t^c^A(G)^g	55
40	18/18_2′-OMe_e50+16	C(M)^U(M)^U(M)^C(M)^C(M)^A(M)^C(M)^U(M)^C(M)^A(M)^ G(M)^A(M)^G(M)^C(M)^U(M)^C(M)^A(M)^G	66
41	15/15_2′-OMe _e50+16	C(M)^C(M)^A(M)^C(M)^U(M)^C(M)^A(M)^G(M)^A(M)^G(M)^ C(M)^U(M)^C(M)^A(M)^G	62
42	13/13_2′-OMe _e50+16	A(M)^C(M)^U(M)^C(M)^A(M)^G(M)^A(M)^G(M)^C(M)^U(M)^ C(M)^A(M)^G	57

Supplementary Table S4. Complementary RNA used for UV melting analysis of SSOs targeting *DMD* exon 50. Sequences are shown from 5′ to 3′.

Entry	Name	Sequence
C-4	Comp. RNA_18/18_RNA_e50+16	CUGAGCUCUGAGUGGAAG
C-5	Comp. RNA_15/15_RNA_e50+16	CUGAGCUCUGAGUGG
C-6	Comp. RNA_13/13_RNA_e50+16	CUGAGCUCUGAGU

Supplementary Table S5. 21-mer BNA-based SSOs targeting *DMD* exon 50 used for the experiment. Seven SSOs for *DMD* exon 50 skipping are shown. Sequences are shown from 5′ to 3′. Lowercase letter: DNA, uppercase character or 5 with (L): LNA, uppercase character or 5 with (A): AmNA, uppercase character or 5 with (G): GuNA, uppercase character with (M): 2′-OMe RNA, 5: 5-methylcytosine, and ^: phosphorothioate.

SSO	Name	Sequence	T_m value (°C)
43	10/21_LNA_e50+16	c^G(L)^c^5(L)^t^T(L)^c^5(L)^a^5(L)^t^5(L)^a^G(L)^a^G(L)^c^ T(L)^c^A(L)^g	88
44	10/21_AmNA_e50+16	c^G(A)^c^5(A)^t^T(A)^c^5(A)^a^5(A)^t^5(A)^a^G(A)^a^G(A)^c^ T(A)^c^A(A)^g	88
45	10/21_GuNA_e50+16	c^G(G)^c^5(G)^t^T(G)^c^5(G)^a^5(G)^t^5(G)^a^G(G)^a^G(G)^ c^T(G)^c^A(G)^g	86
46	7/21_LNA_e50+16	c^G(L)^c^c^T(L)^t^c^5(L)^a^c^T(L)^c^a^G(L)^a^g^5(L)^t^c^ A(L)^g	75
47	7/21_AmNA_e50+16	c^G(A)^c^c^T(A)^t^c^5(A)^a^c^T(A)^c^a^G(A)^a^g^5(A)^t^c^	74

		A(A)^g	
48	7/21_GuNA_e50+16	c^G(G)^c^c^T(G)^t^c^5(G)^a^c^T(G)^c^a^G(G)^a^g^5(G)^t^c^<br>A(G)^g	74
49	21/21_2′-OMe_e50+16	C(M)^G(M)^C(M)^C(M)^U(M)^U(M)^C(M)^C(M)^A(M)^C(M)^<br>U(M)^C(M)^A(M)^G(M)^A(M)^G(M)^C(M)^U(M)^C(M)^A(M)^G	69

Supplementary Table S6. Complementary RNA used for UV melting analysis of 21-mer SSOs targeting *DMD* exon 50. Sequence is shown from 5′ to 3′.

Entry	Name	Sequence
C-7	Comp. RNA_21/21_RNA_e50+16	CUGAGCUCUGAGUGGAAGGCG

Supplementary Table S7. Primers used for RT-PCR analysis investigating *DMD* exon 50 skipping. Sequences of forward (For) and reverse (Rev) primers for each target are shown. Sequences are shown from 5′ to 3′.

Gene		Sequence	Size （bp）
DMD	For primer	tctgctgctgtggttatctcc	455
	Rev primer	aagccgagtgacattctggg	346 (exon 50 skipped)<br>334 (exon 58 skipped)
GADPH	For primer	accacagtccatgccatcac	452
	Rev primer	tccaccaccctgttgctgta	

Supplementary Table S8. Primers used for quantitative PCR analysis investigating *DMD* exon 58 skipping. Sequences of forward (For) and reverse (Rev) primers for each target are shown. Sequences are shown from 5′ to 3′. In details, please see our previous report.[6]

Gene		Sequence	Size （bp）
DMD	For primer<br>Rev primer	agttctgaccagtggaagcg<br>cctcaggaggcagctcctat	156
RPLP2	For primer<br>Rev primer	tggacagcgtgggtatcgag<br>ctgggcaatgacgtcttcaa	92

Supplementary Table S9. Primers used for quantitative PCR analysis investigating *DMD* exon 50 skipping. Sequences of forward (For) and reverse (Rev.) primers for each target are shown. Sequences are shown from 5′ to 3′. We designed the specific primers for detecting *DMD* exon 50 skipping according to our previous report.[5] In details, For. primer hybridizes with the exon 49, and Rev. primer hybridizes with exon junction between exon 49 and exon 51. The expression of human *RPLP2* mRNA was used to normalize the data.

Gene		Sequence	Size (bp)
DMD	For primer	acaaccggatgtggaagagatt	95 bp
	Rev primer	gtaacagtctgagtaggagcttca	
RPLP2	For primer	tggacagcgtgggtatcgag	92 bp
	Rev primer	ctgggcaatgacgtcttcaa	

Supplementary Table S10. LNA-based SSOs used for target selection of *DMD* exon 50. Thirteen SSOs for *DMD* exon 50 skipping are shown. Sequences are shown from 5′ to 3′. Lowercase letter: DNA, uppercase character or 5 with (L): LNA, uppercase character with (M): 2′-OMe RNA, 5: 5-methylcytosine, and ^: phosphorothioate.

SSO	Name	Sequence
50	7/15_LNA_e50+4	c^A(L)^g^A(L)^t^5(L)^t^T(L)^c^T(L)^a^A(L)^c^T(L)^t
51	7/15_LNA_e50+8	a^G(L)^c^T(L)^c^A(L)^g^A(L)^t^5(L)^t^T(L)^c^T(L)^a
52	7/15_LNA_e50+12	t^5(L)^a^G(L)^a^G(L)^c^T(L)^c^A(L)^g^A(L)^t^5(L)^t
28	7/15_LNA_e50+16	c^5(L)^a^5(L)^t^5(L)^a^G(L)^a^G(L)^c^T(L)^c^A(L)^g
53	7/15_LNA_e50+20	c^5(L)^t^T(L)^c^5(L)^a^5(L)^t^5(L)^a^G(L)^a^G(L)^c
54	7/15_LNA_e50+83	t^G(L)^g^T(L)^c^A(L)^g^T(L)^c^5(L)^a^G(L)^g^A(L)^g
55	7/15_LNA_e50+87	a^T(L)^a^G(L)^t^G(L)^g^T(L)^c^A(L)^g^T(L)^c^5(L)^a

56	7/15_LNA_e50+91	t^5(L)^c^A(L)^a^T(L)^a^G(L)^t^G(L)^g^T(L)^c^A(L)^g
57	7/15_LNA_e50+95	a^G(L)^g^5(L)^t^5(L)^c^A(L)^a^T(L)^a^G(L)^t^G(L)^g
58	7/15_LNA_e50+99	t^T(L)^a^5(L)^a^G(L)^g^5(L)^t^5(L)^c^A(L)^a^T(L)^a
59	7/15_LNA_e50+103	a^T(L)^a^5(L)^t^T(L)^a^5(L)^a^G(L)^g^5(L)^t^5(L)^c
60	7/15_LNA_e50+107	c^A(L)^g^T(L)^a^T(L)^a^5(L)^t^T(L)^a^5(L)^a^G(L)^g
61	15/15_2′-OMe_e50+11	C(M)^U(M)^C(M)^A(M)^G(M)^A(M)^G(M)^C(M)^U(M)^C(M)^A(M)^G(M)^A(M)^U(M)^ C(M)^U(M)^U(M)

Supplementary Figure S1. UV melting analysis for 18-mer LNA/AmNA/GuNA-modified SSOs targeting *DMD* exon 58 having different modification content. a) and c) Results of UV melting experiments for single-stranded LNA-, AmNA-, and GuNA-modified SSOs having high modification contents. b) and d) Results of UV melting experiments for single-stranded LNA-, AmNA-, and GuNA-modified SSOs having low modification contents. All analyses were repeated three times to ensure reproducibility. These data are reproducible results in Figure 3. The relative UV absorbance depicts normalized data (normalized by UV absorption at 94.5 °C set to 1). e)-j) Derivative of Figures 3a, 3b and Supplementary Figures S1a-d respectively.

Supplementary Fig.1

a n=2

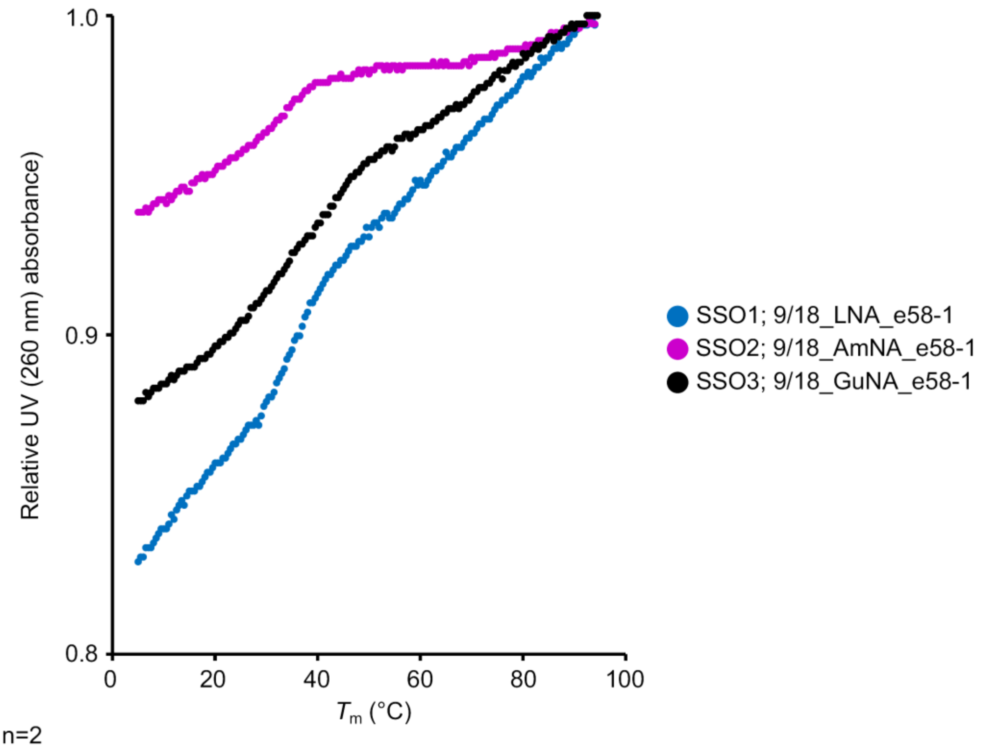

b n=2

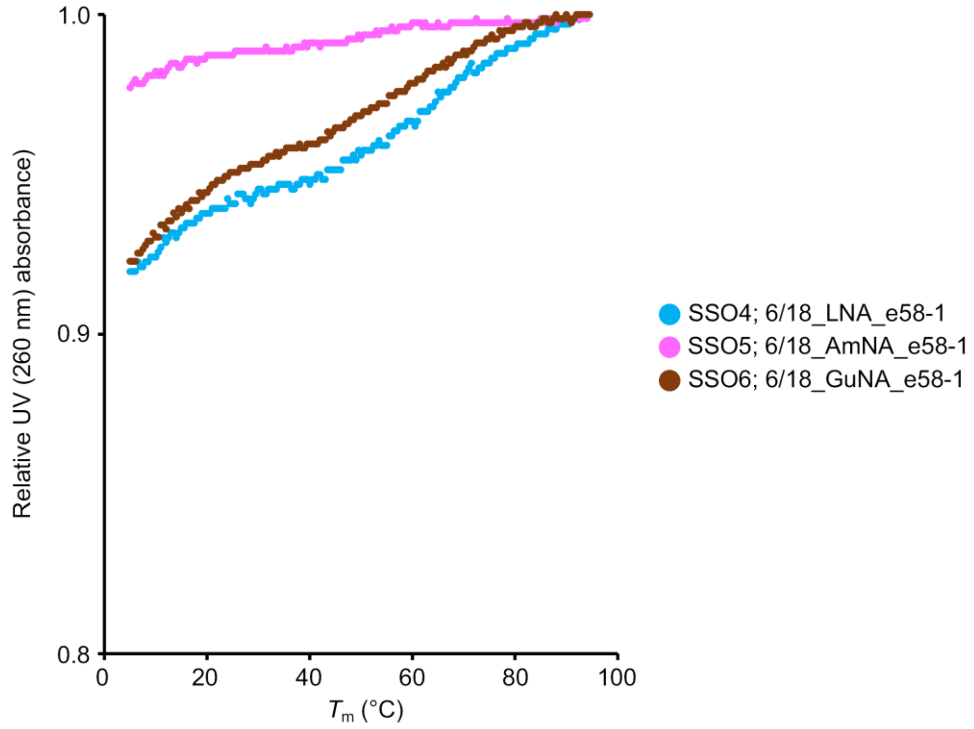

Supplementary Fig.1 (continued)

c n=3

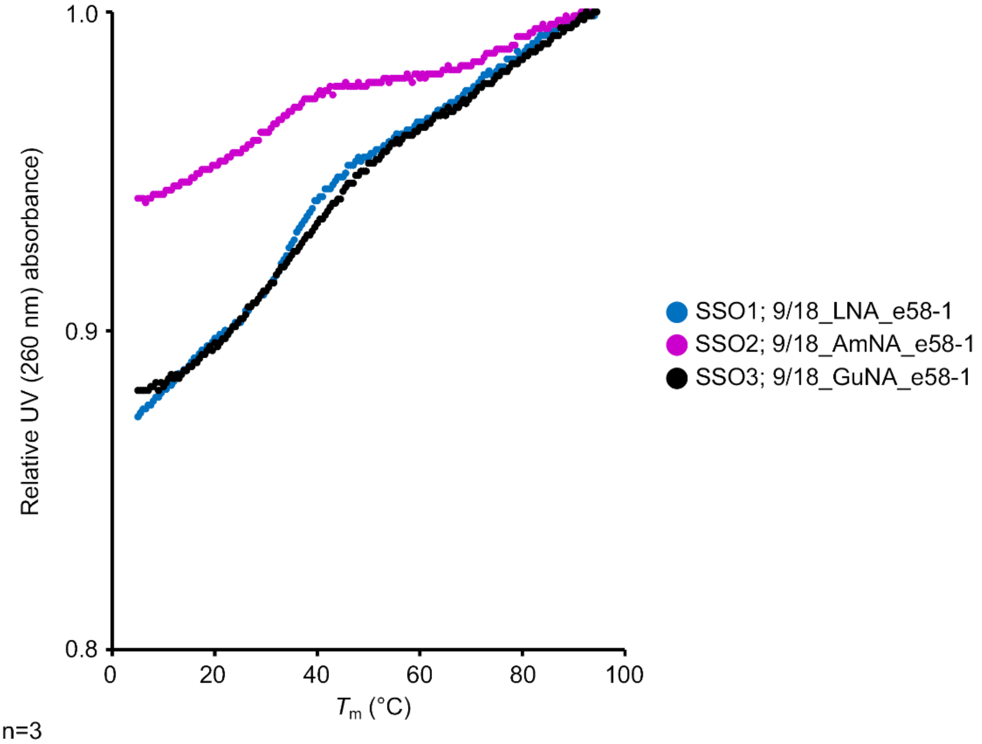

d n=3

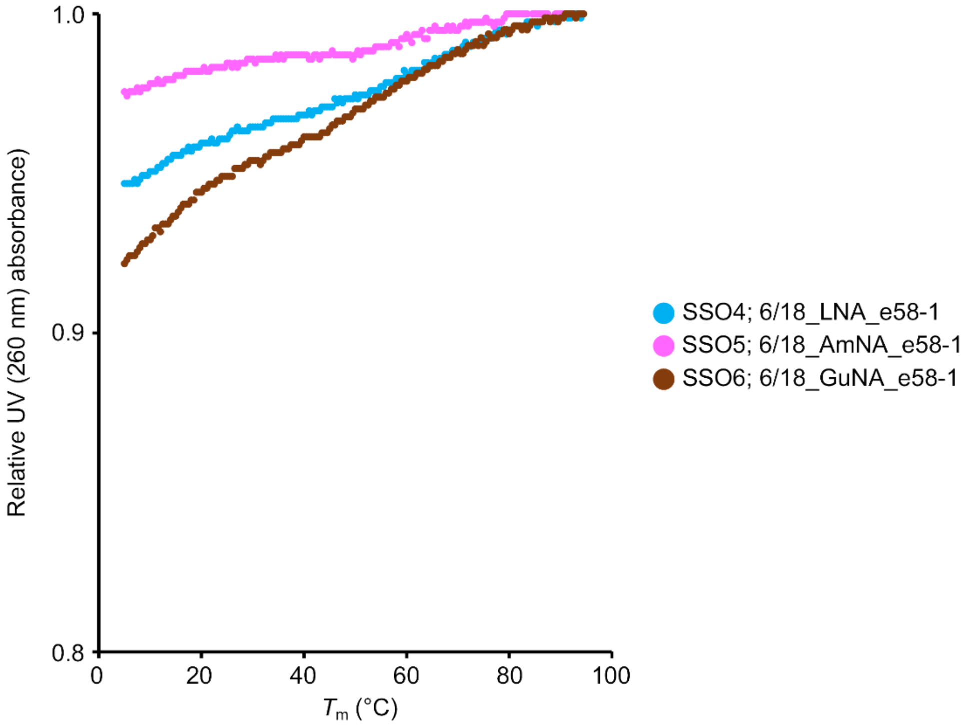

Supplementary Fig.1 (continued)

e n=1

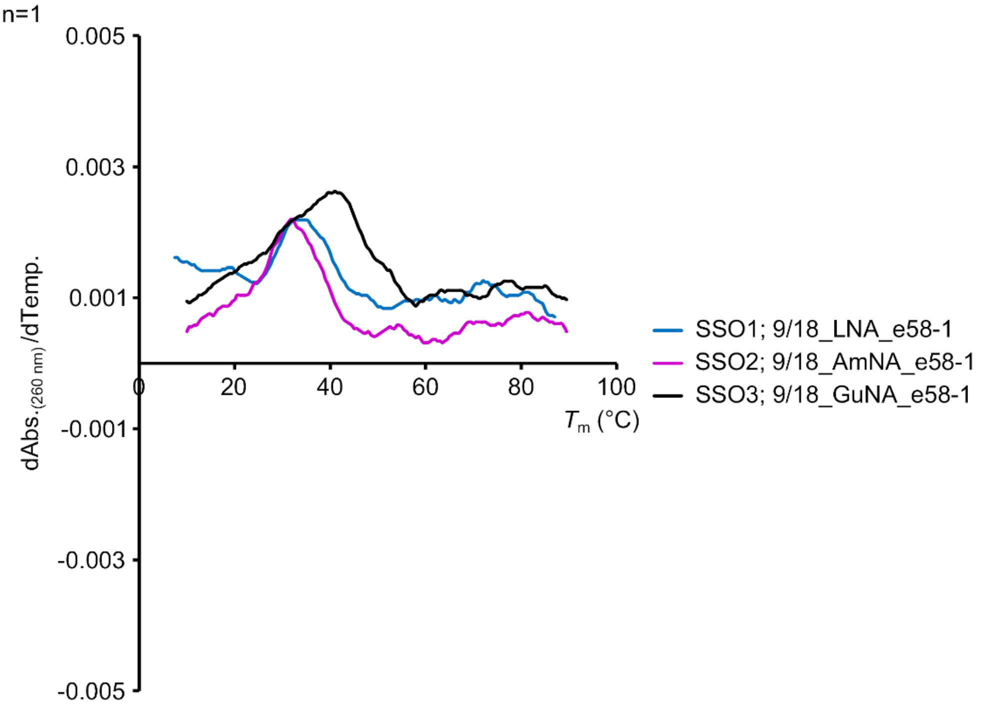

f n=1

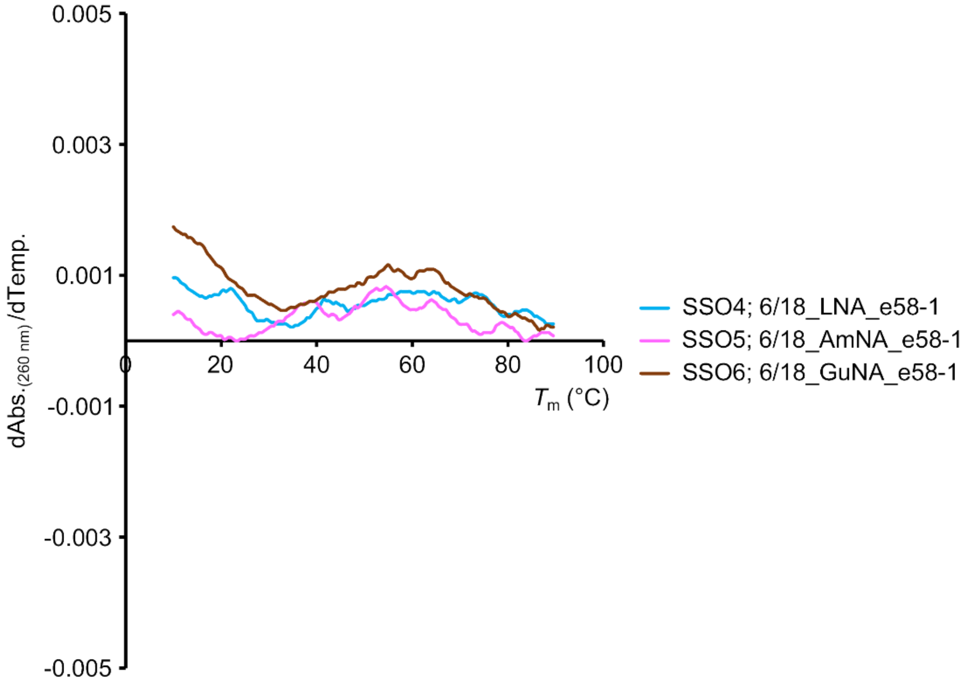

Supplementary Fig.1 (continued)

g n=2

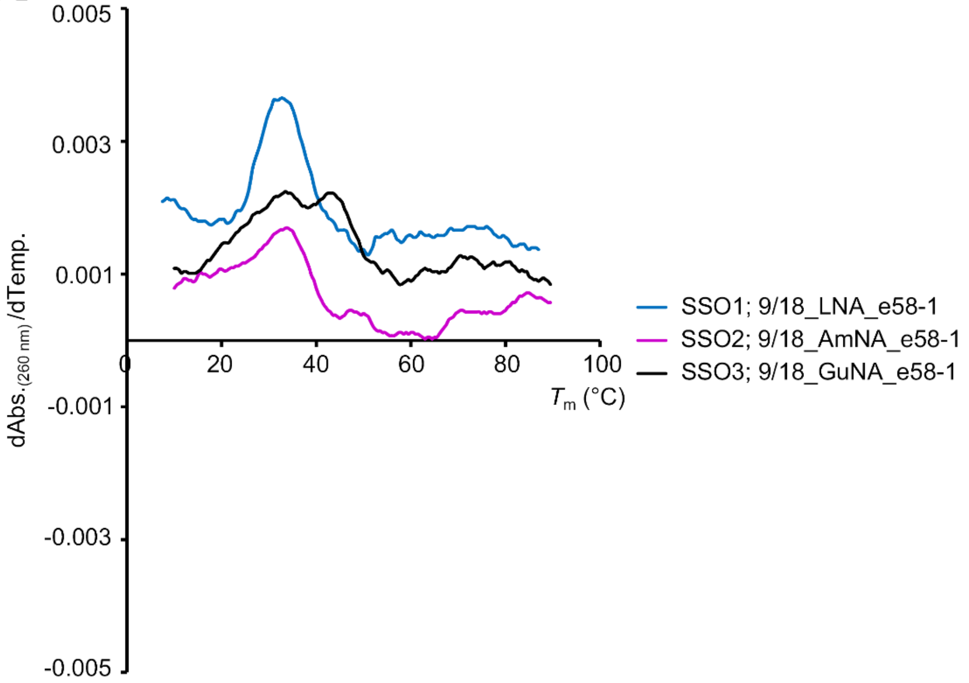

h n=2

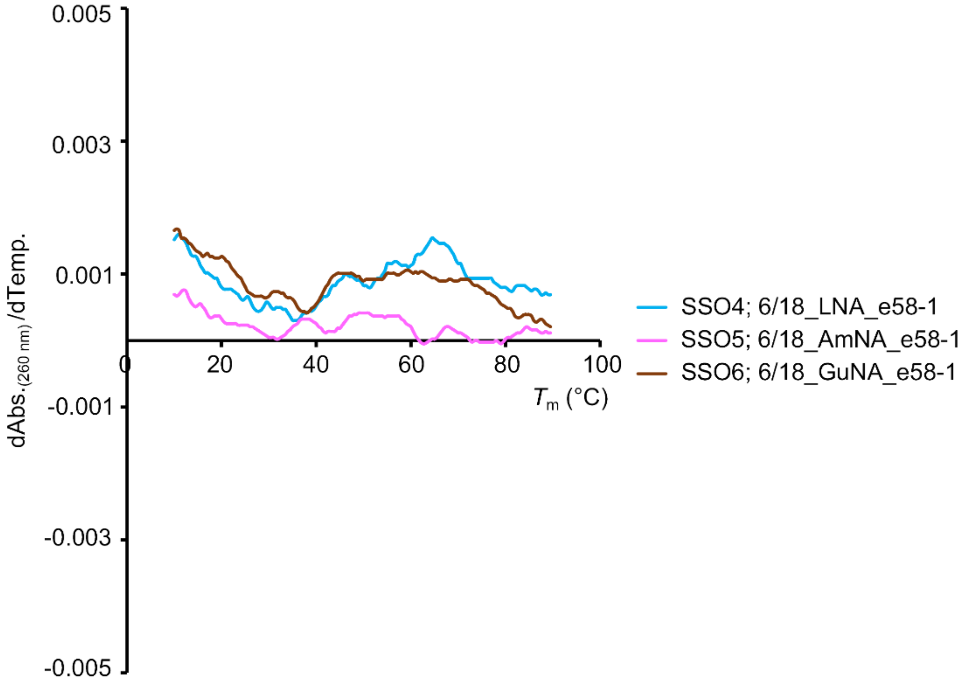

Supplementary Fig.1 (continued)

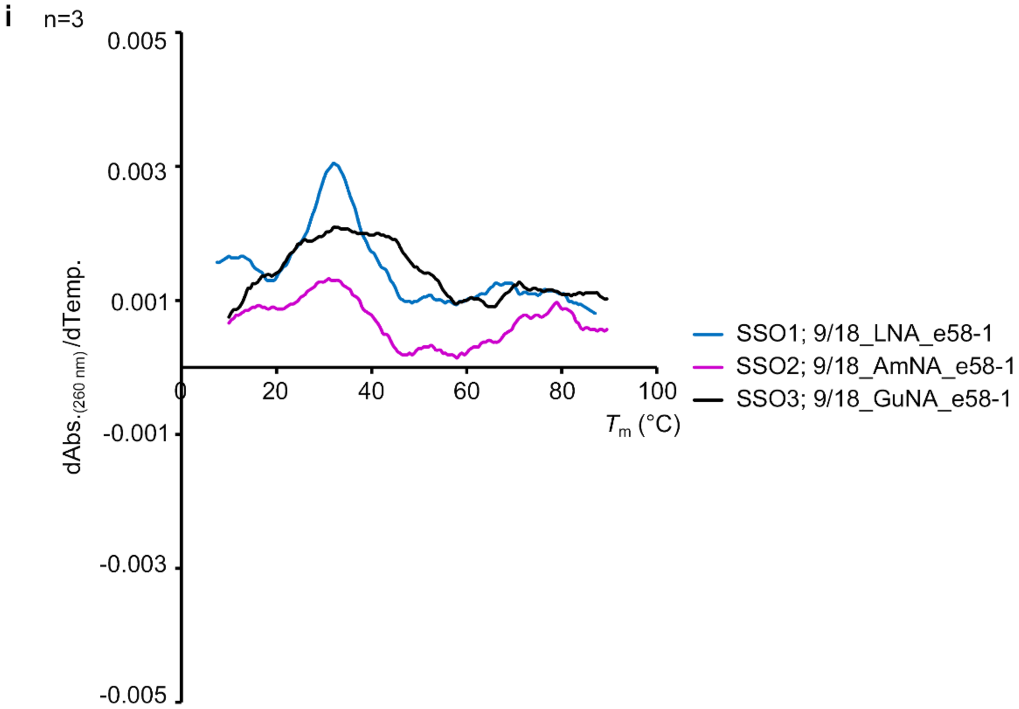

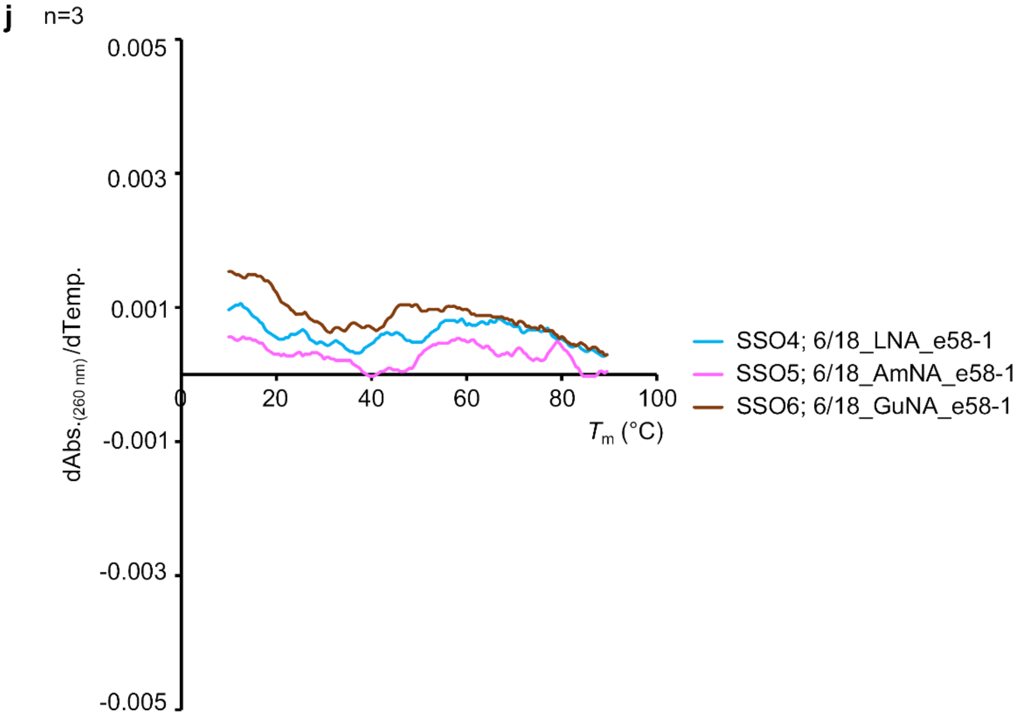

Supplementary Figure S2. Screening of 15-mer LNA-modified SSOs targeting *DMD* exon 50 using the DMD model cell line.

a) Schematic representation of SSOs used for screening the target sites on *DMD* exon 50 (also see Supplementary Table S10). Entries 50–61 show LNA-modified SSOs, and entry 62 shows the 2′-OMe RNA-modified SSO, h50AON1, named by Aartsma-Rus *et al.*[2] b) Schematic representation of target sites for each SSO. c) Results of RT-PCR analysis. The DMD model cells were transfected with the indicated SSOs (500 nM) for 24 h. RT-PCR shows the full-length upper band (455-bp) and skipped lower band (346-bp). *GADPH* was used as an internal control. Mock: treated with Lipofectamine only; No treatment: no transfection.

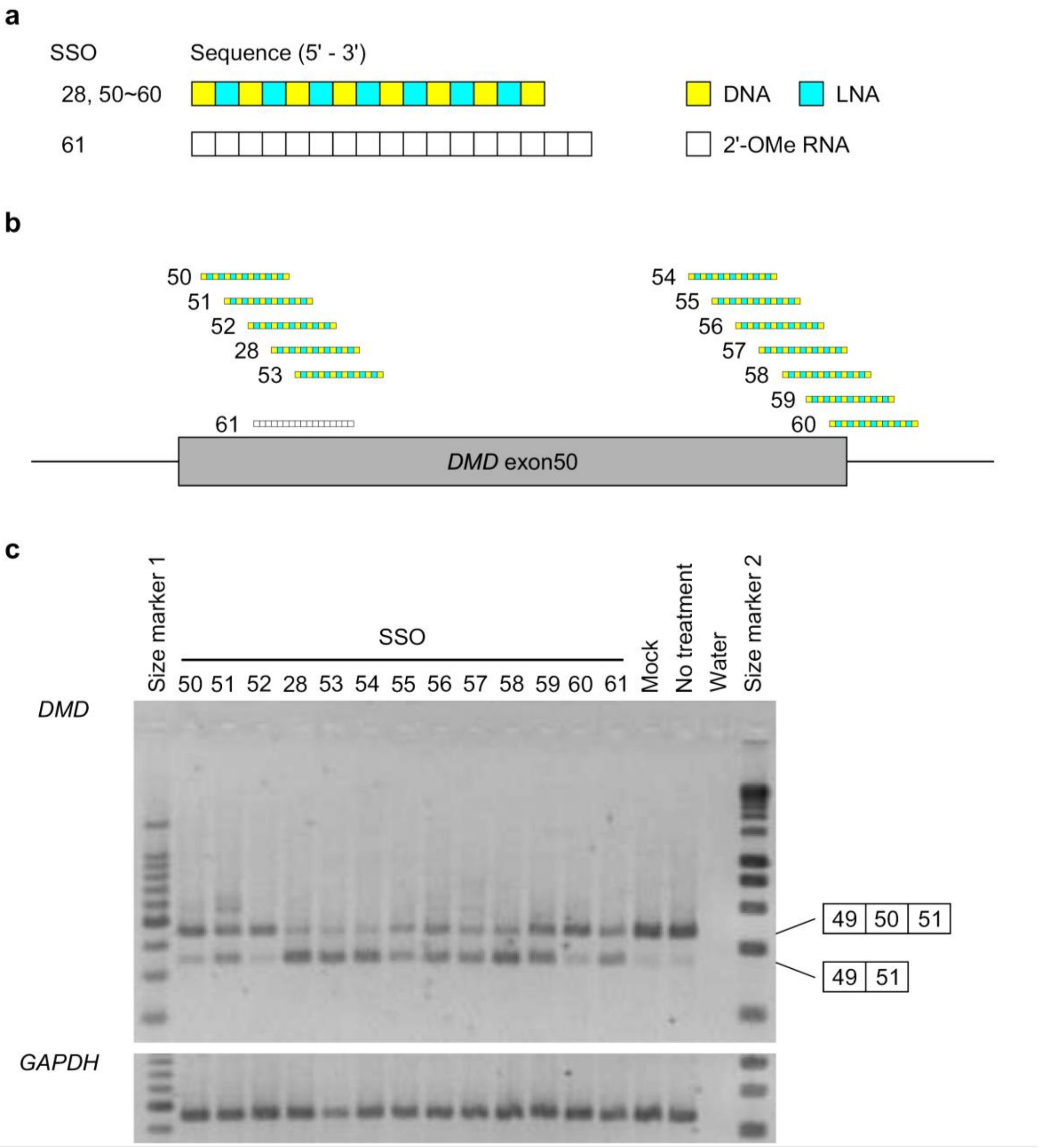
a
SSO
Sequence (5' - 3')
28, 50~60
61
DNA
LNA
2'-OMe RNA
b
50
51
52
28
53
61
54
55
56
57
58
59
60
DMD exon50
c
Size marker 1
SSO
50 51 52 28 53 54 55 56 57 58 59 60 61
Mock
No treatment
Water
Size marker 2
DMD
GAPDH
49 50 51
49 51

Supplementary Figure S3. Schematic representation of the DMD minigene and its splicing pattern.

Human dystrophin exons are indicated by gray boxes and introns by narrow horizontal black lines. The black boxes represent vector sequences. The expected mRNA structures, indicated below the minigene structure result from the inclusion or exclusion of exon 50.

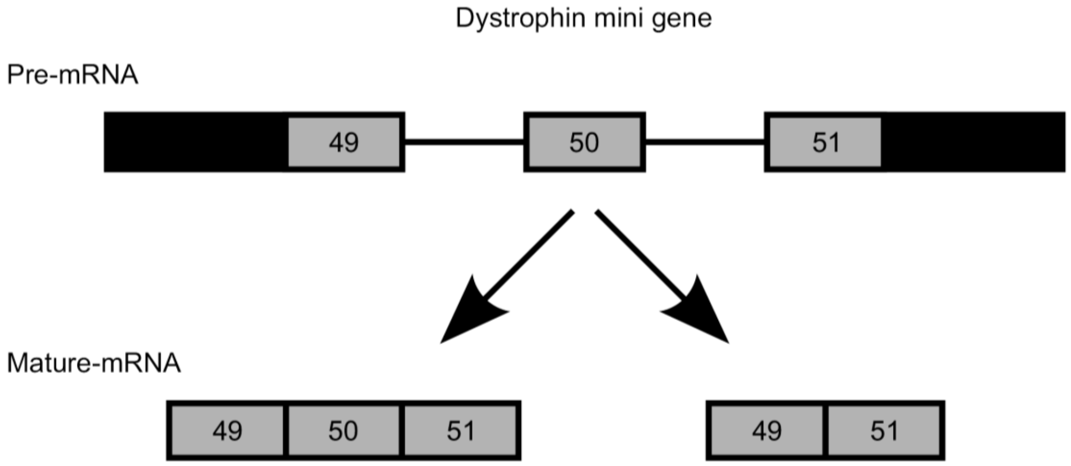

Supplementary Figure S4. UV melting analysis for 18-mer LNA/AmNA/GuNA-modified SSOs targeting *DMD* exon 50 having different modification content. a) and c) Results of UV melting experiments for single-stranded LNA-, AmNA-, and GuNA-modified SSOs having high modification contents. b) and d) Results of UV melting experiments for single-stranded LNA-, AmNA-, and GuNA-modified SSOs having low modification contents. All analyses were repeated three times to ensure reproducibility. These data are reproducible results in Figure 6. The relative UV absorbance depicts normalized data (normalized by UV absorption at 94.5 °C set to 1). e)-j) Derivative of the results of Figures 6a, 6b and Supplementary Figures S4a-d respectively.

Supplementary Fig.4

a n=2

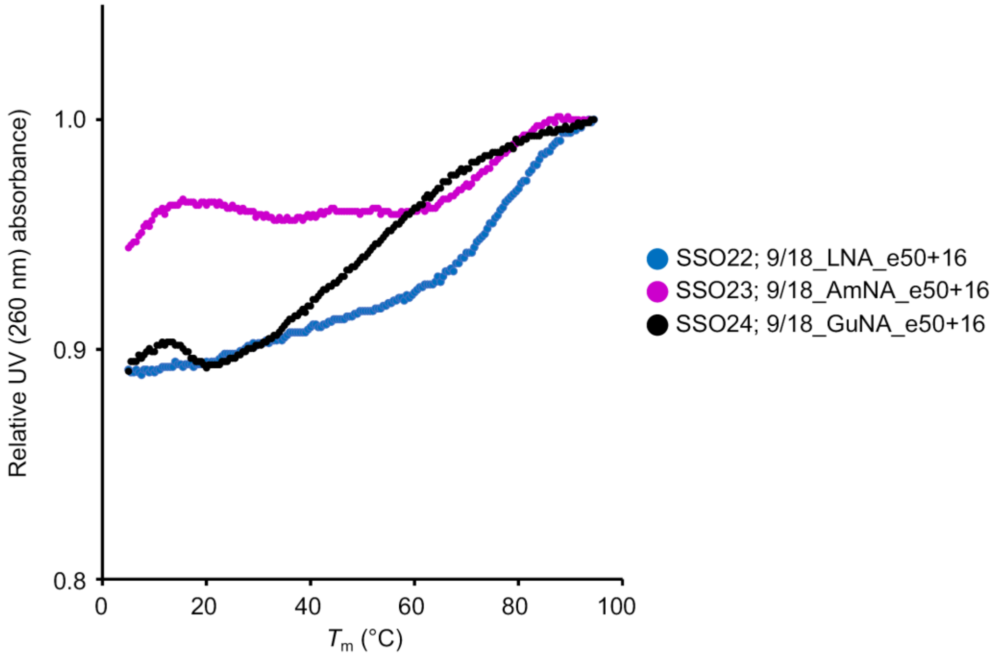

b n=2

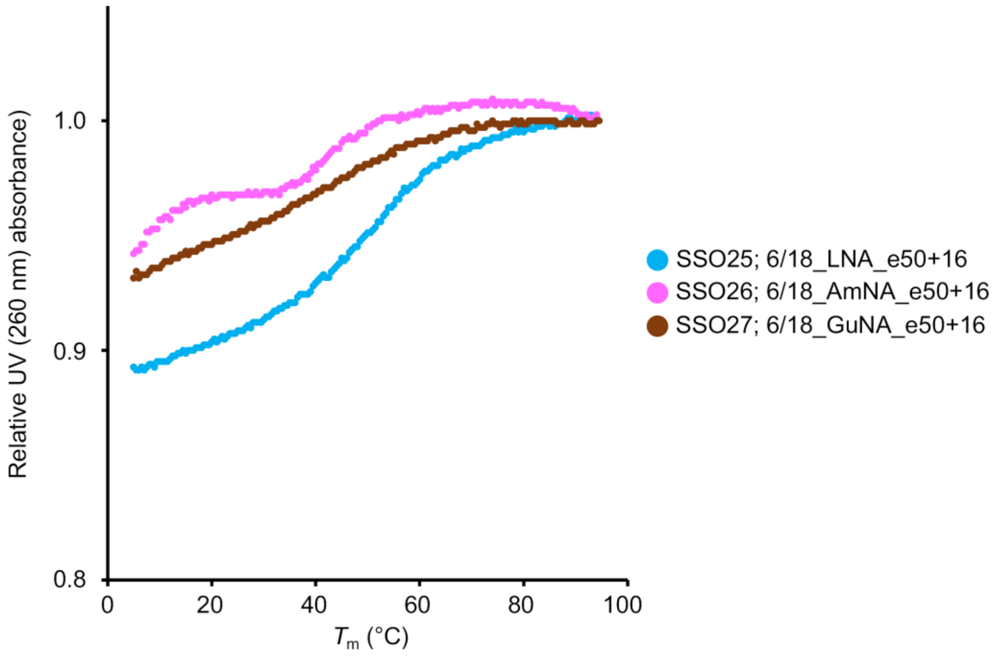

Supplementary Fig.4 (continued)

c n=3

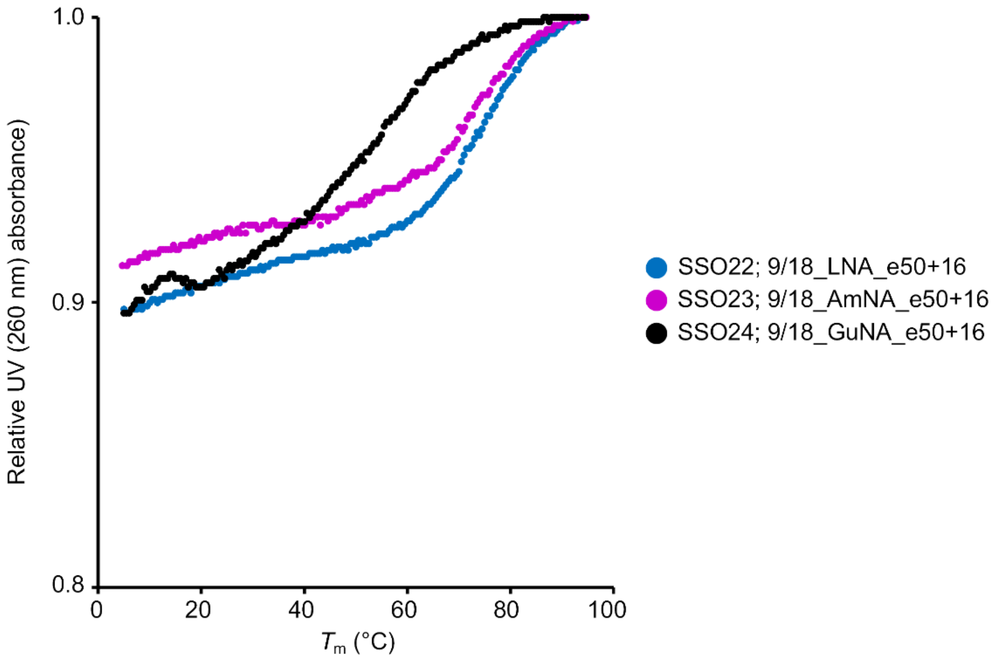

d n=3

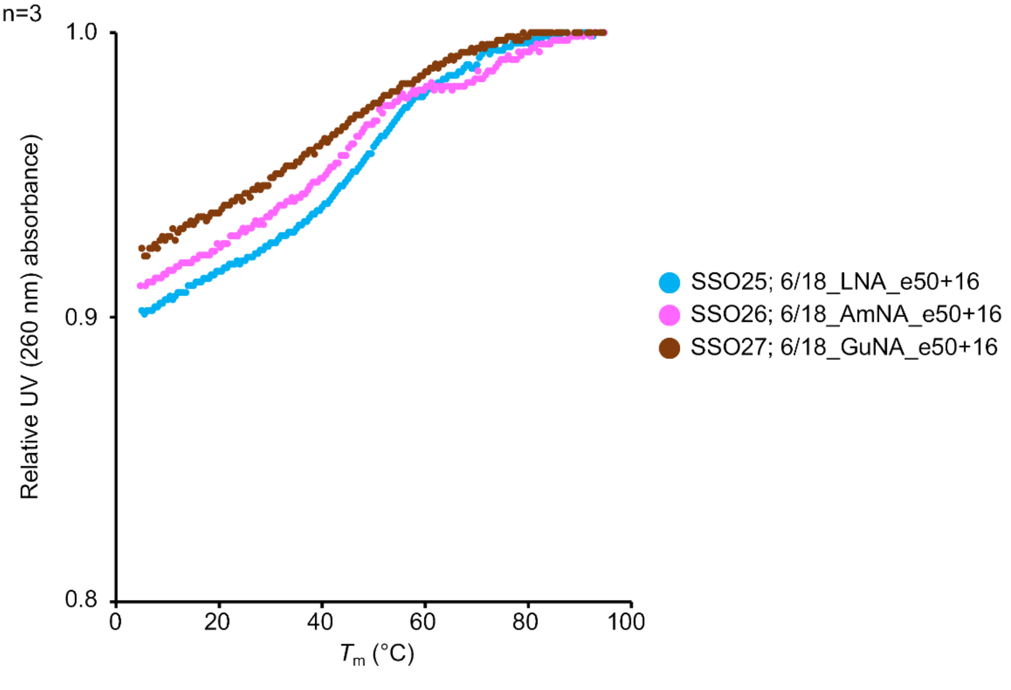

Supplementary Fig.4 (continued)

e n=1

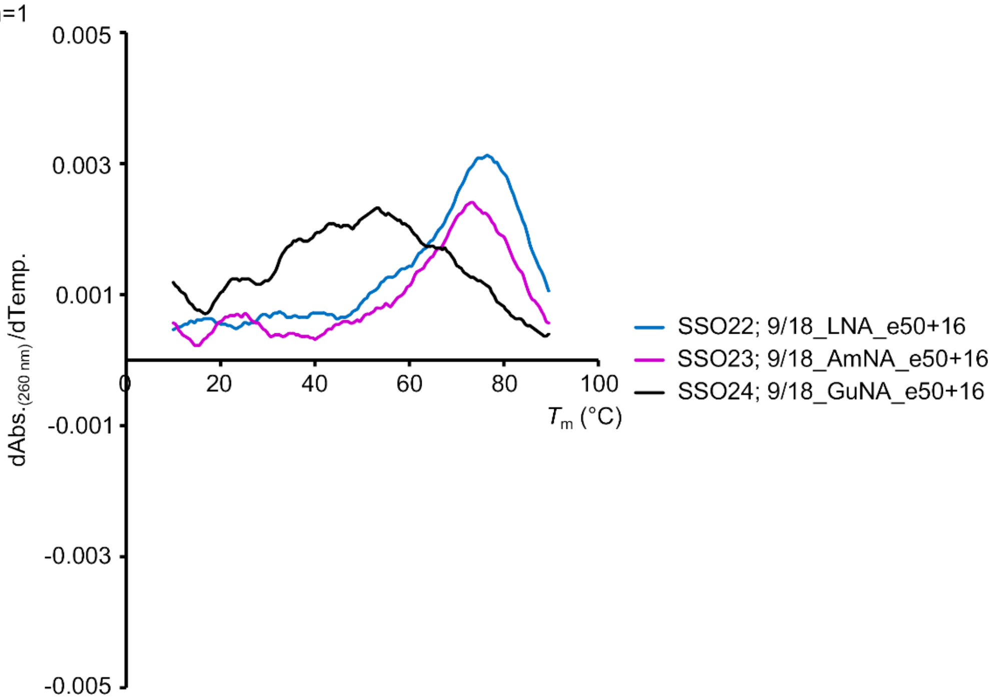

f n=1

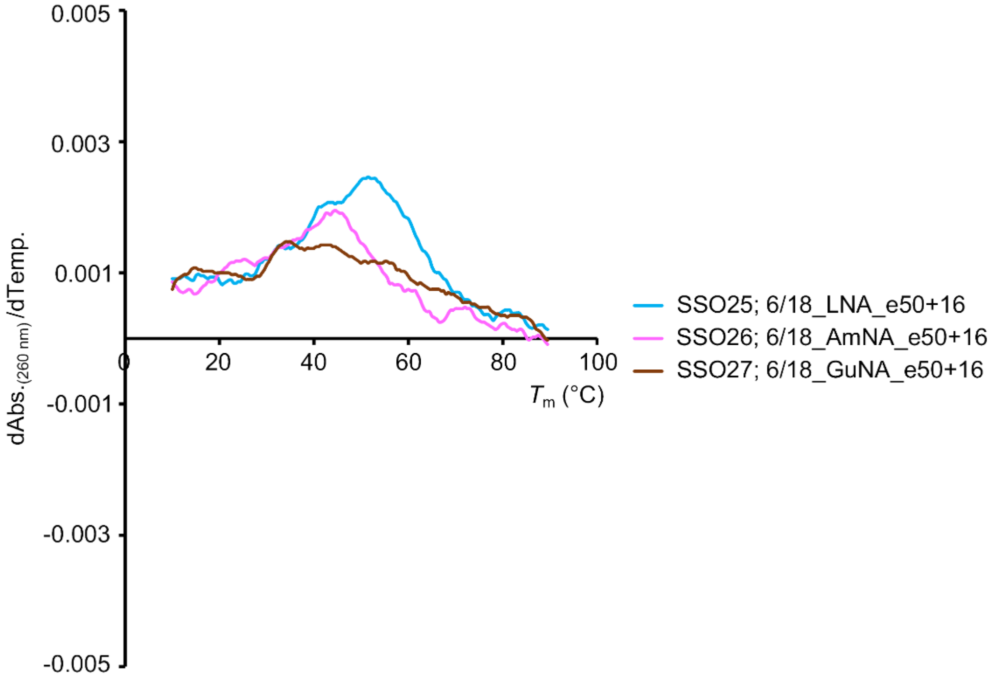

Supplementary Fig.4 (continued)

g n=2

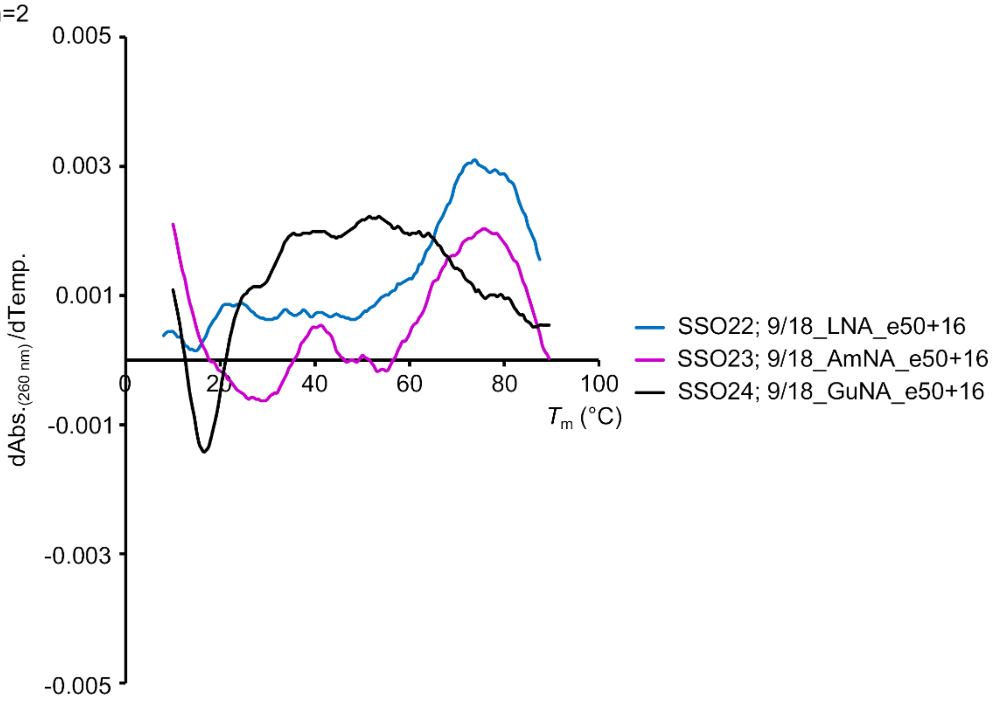

h n=2

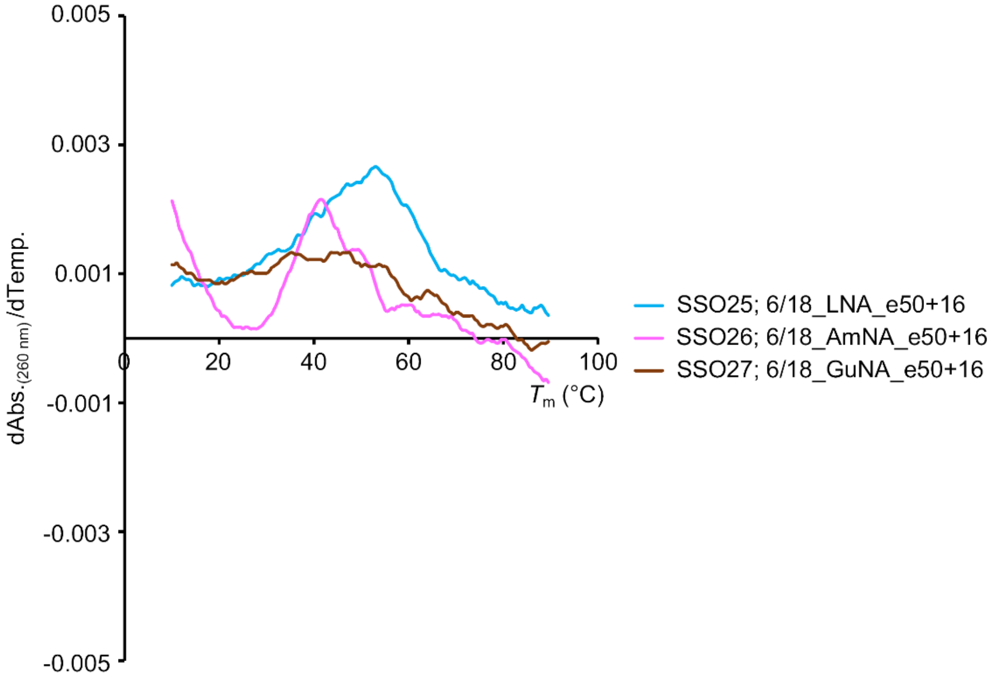

Supplementary Fig.4 (continued)

i

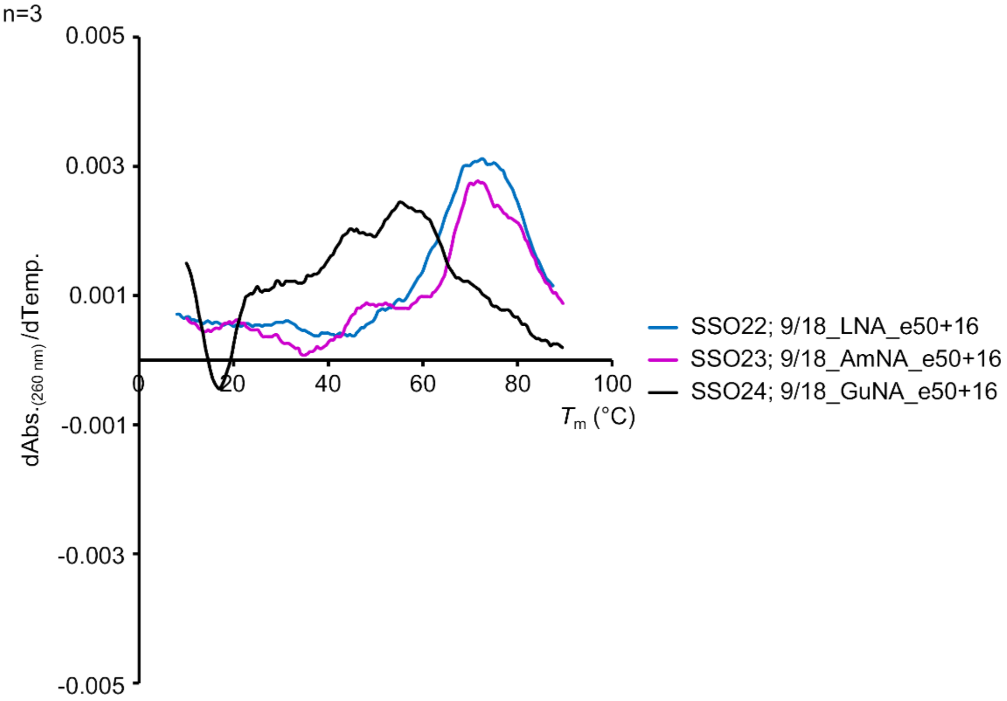

j

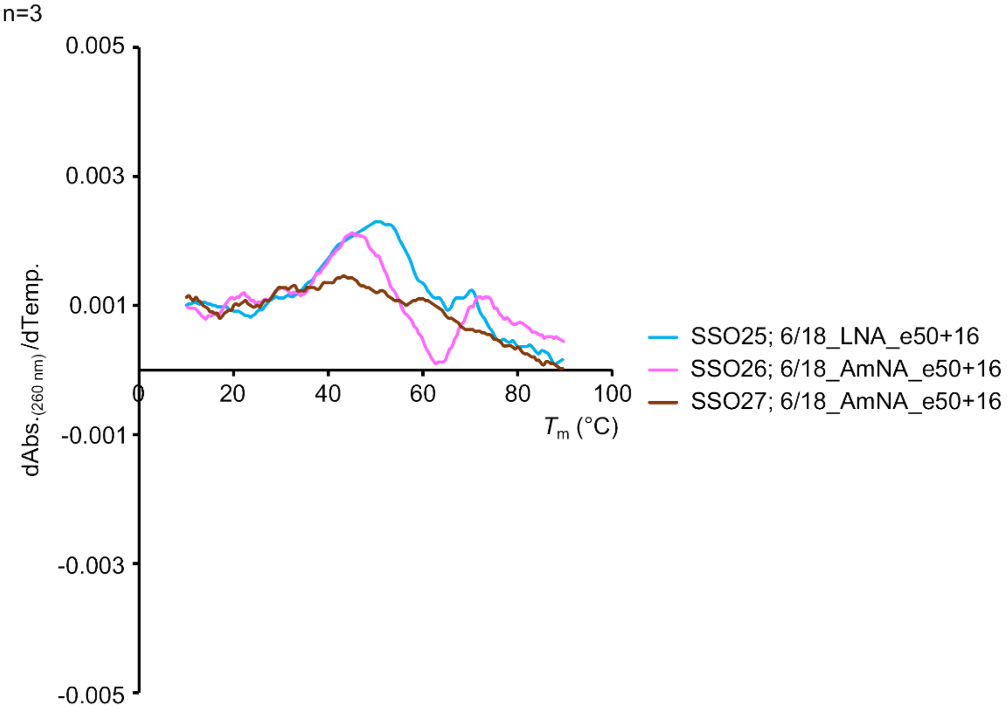

Supplementary Figure S5. UV melting analysis for 21-mer LNA/AmNA/GuNA-modified SSOs targeting *DMD* exon 50 having different modification content. a), c) and e) Results of UV melting experiments for single-stranded LNA-, AmNA-, and GuNA-modified SSOs having high modification contents. b), d) and f) Results of UV melting experiments for single-stranded LNA-, AmNA-, and GuNA-modified SSOs having low modification contents. All analyses were repeated three times to ensure reproducibility. The relative UV absorbance depicts normalized data (normalized by UV absorption at 94.5 °C set to 1). g)-l) Derivative of the results of Supplementary Figures S5a-f respectively.

Supplementary Fig.5

a n=1

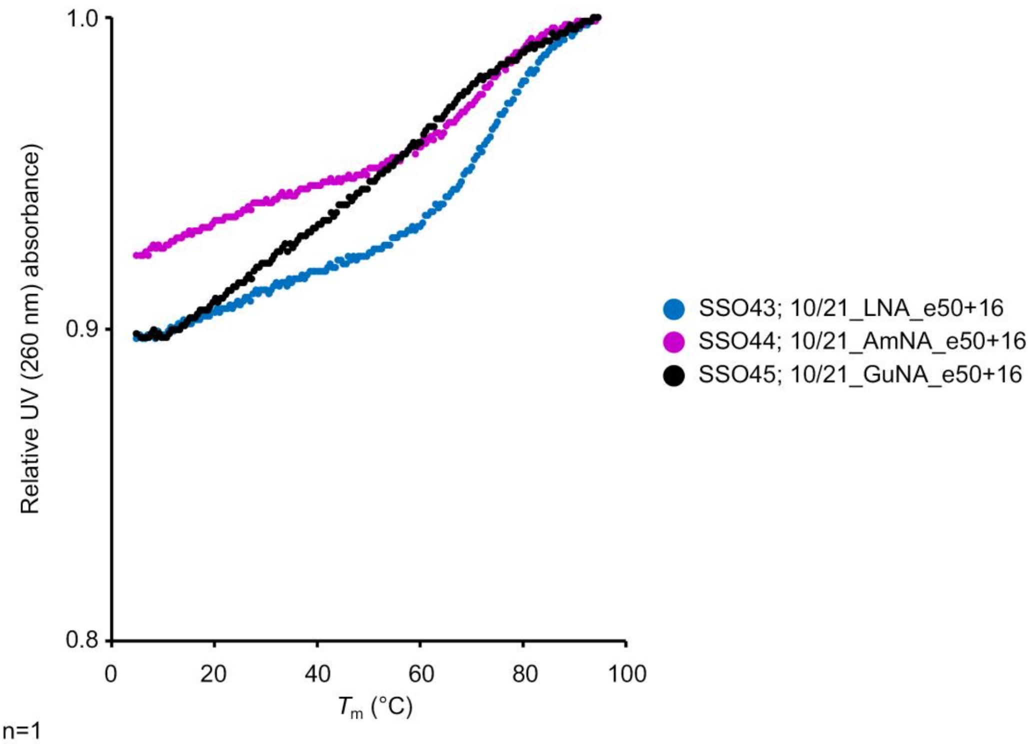

b n=1

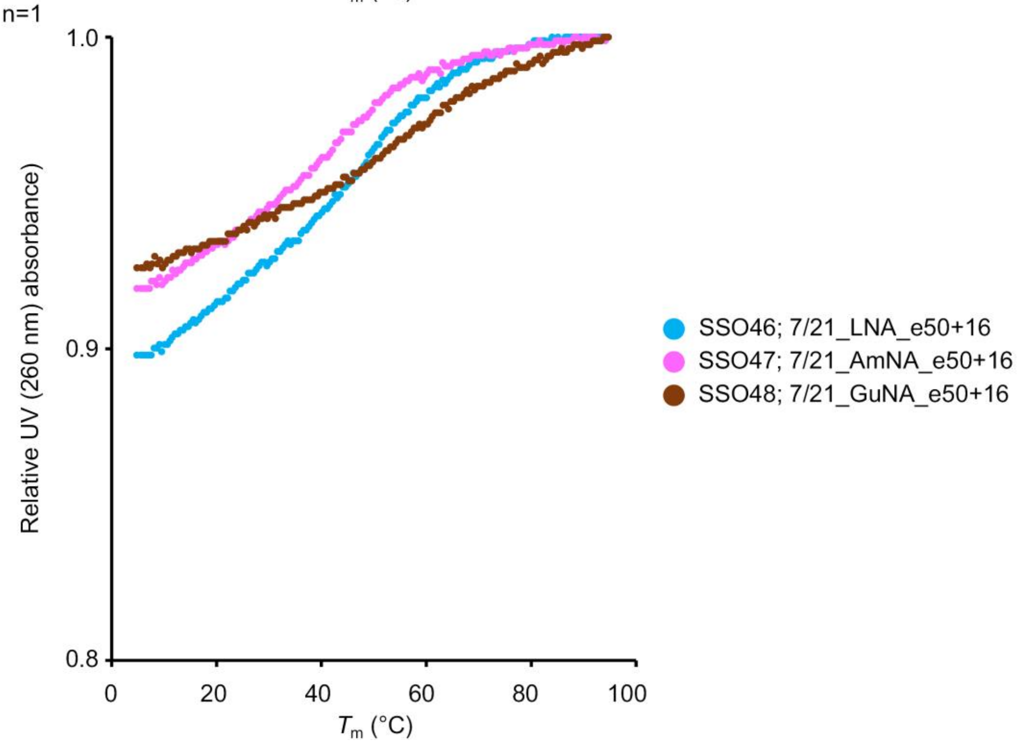

Supplementary Fig.5 (continued)

c n=2

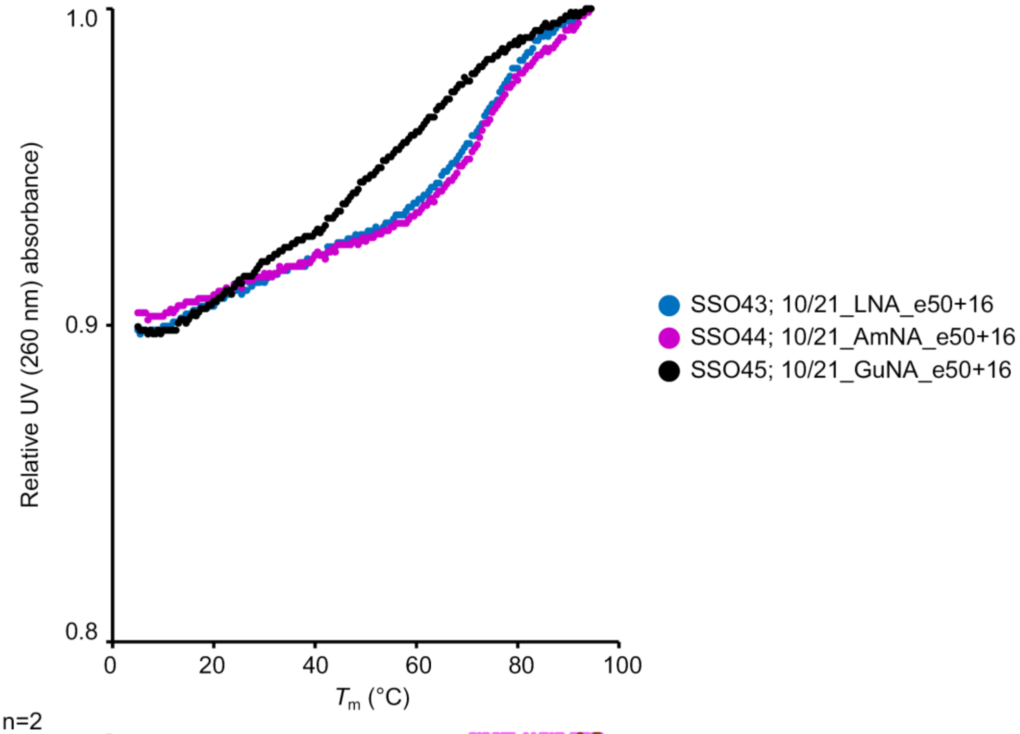

d n=2

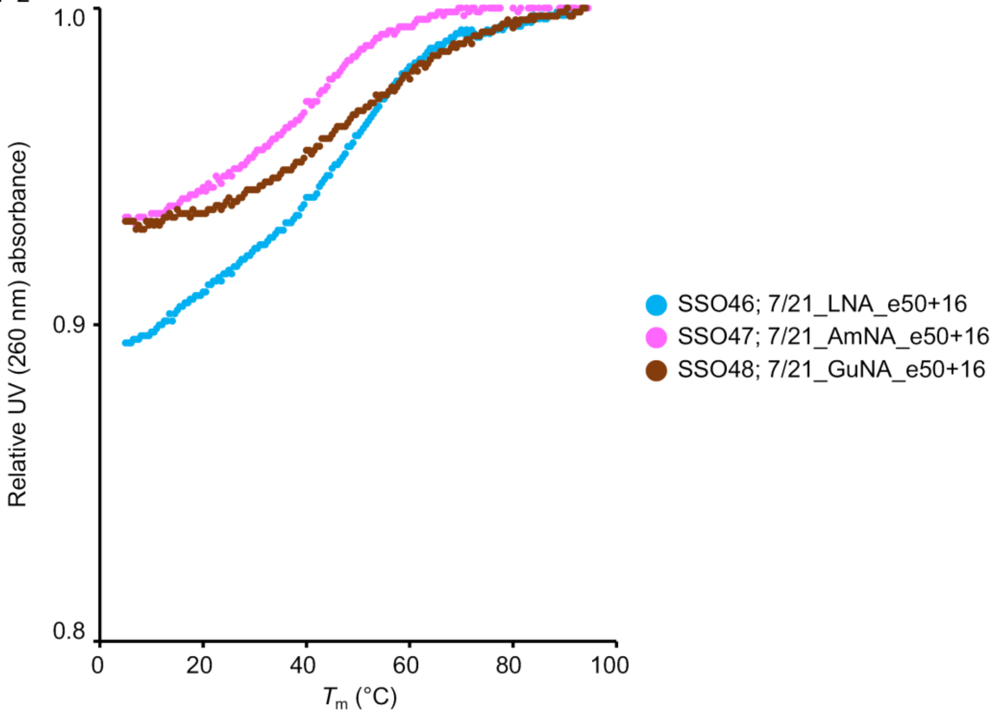

Supplementary Fig.5 (continued)

e n=3

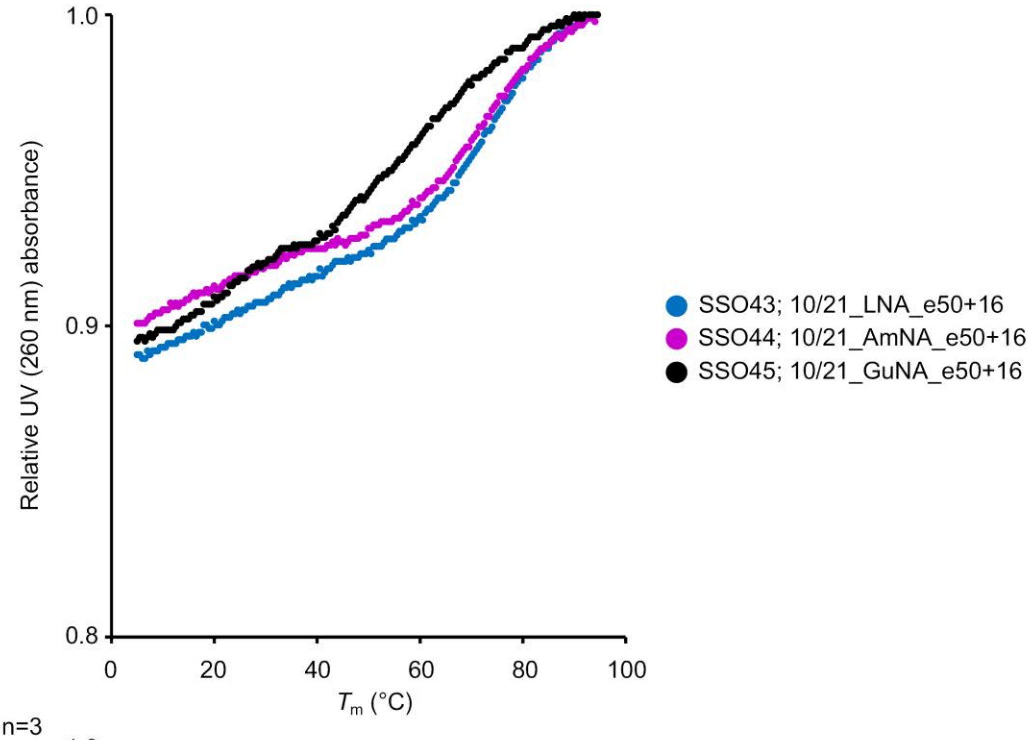

f n=3

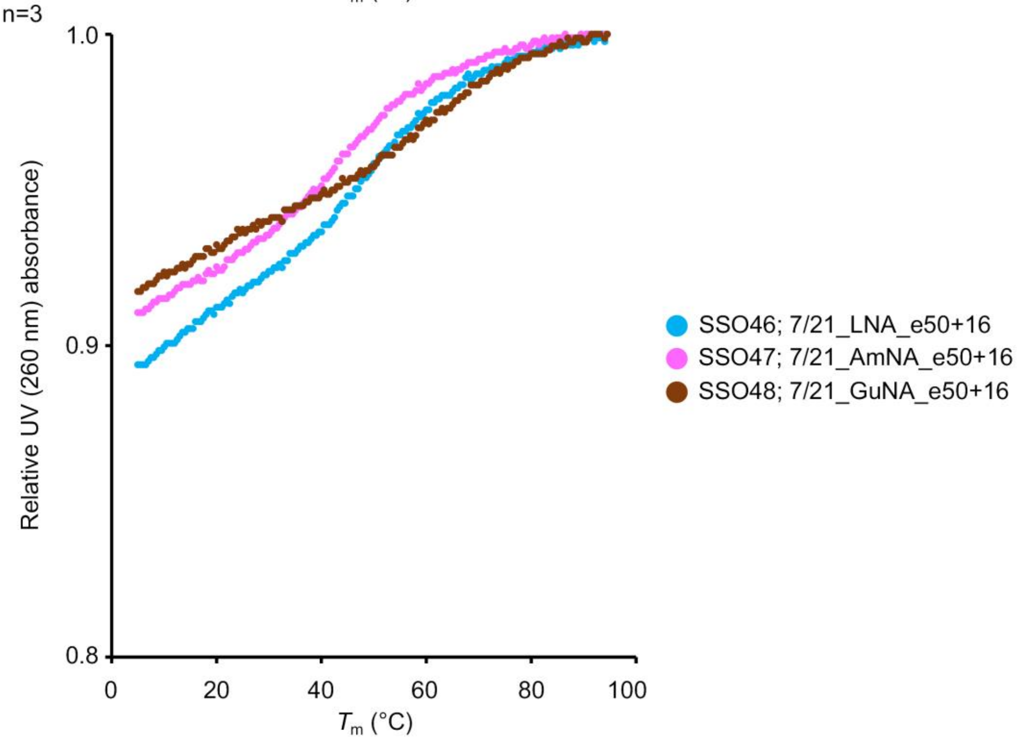

Supplementary Fig.5 (continued)

g n=1

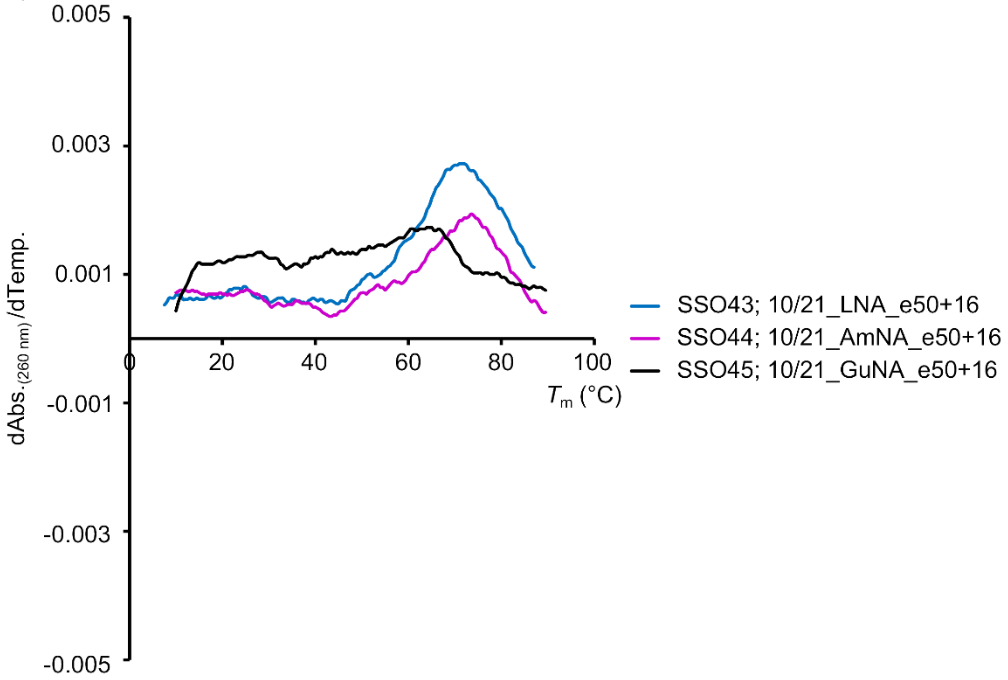

h n=1

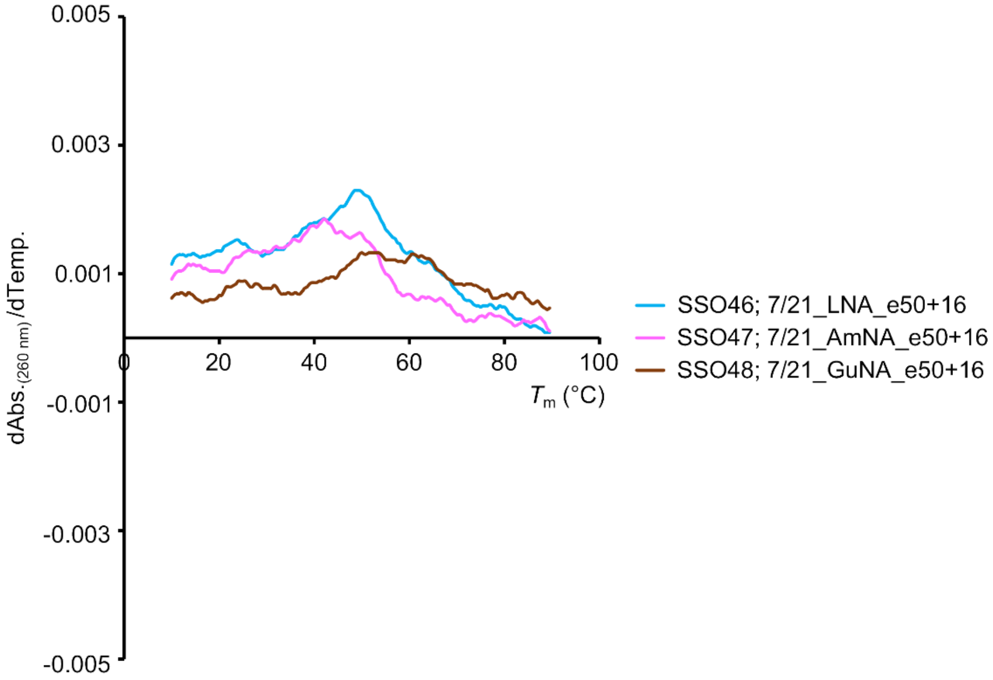

Supplementary Fig.5 (continued)

i n=2

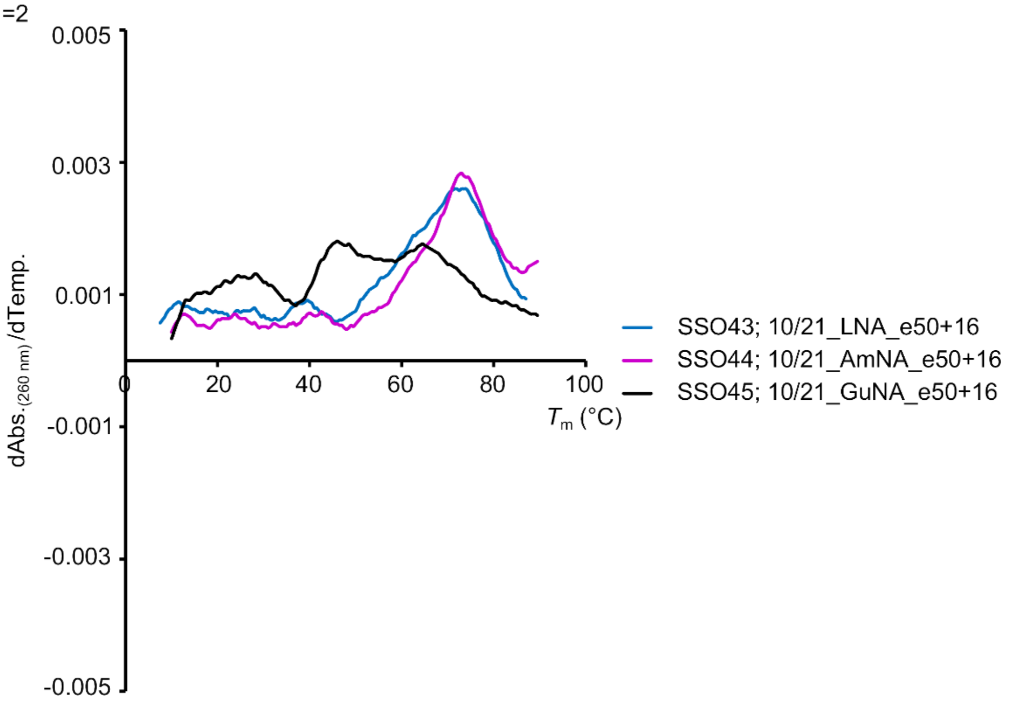

j n=2

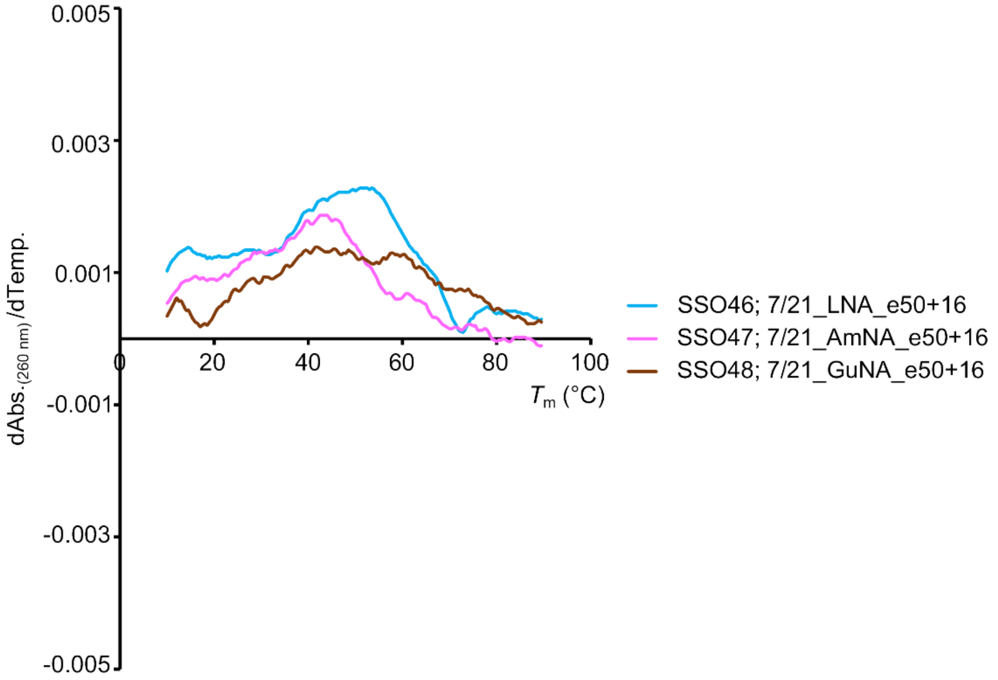

Supplementary Fig.5 (continued)

k n=3

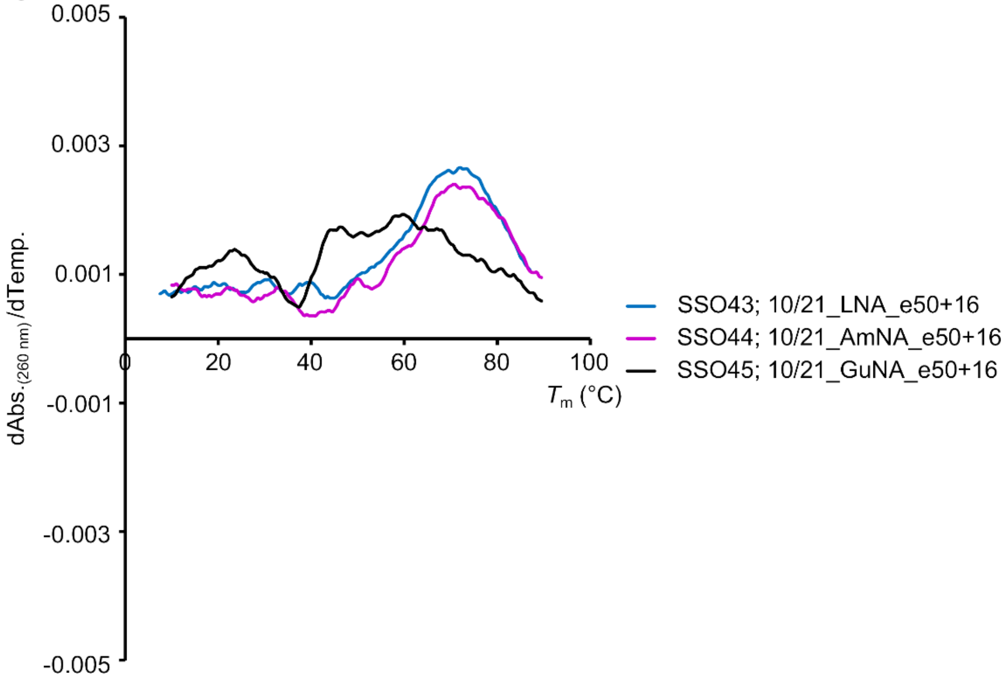

l n=3

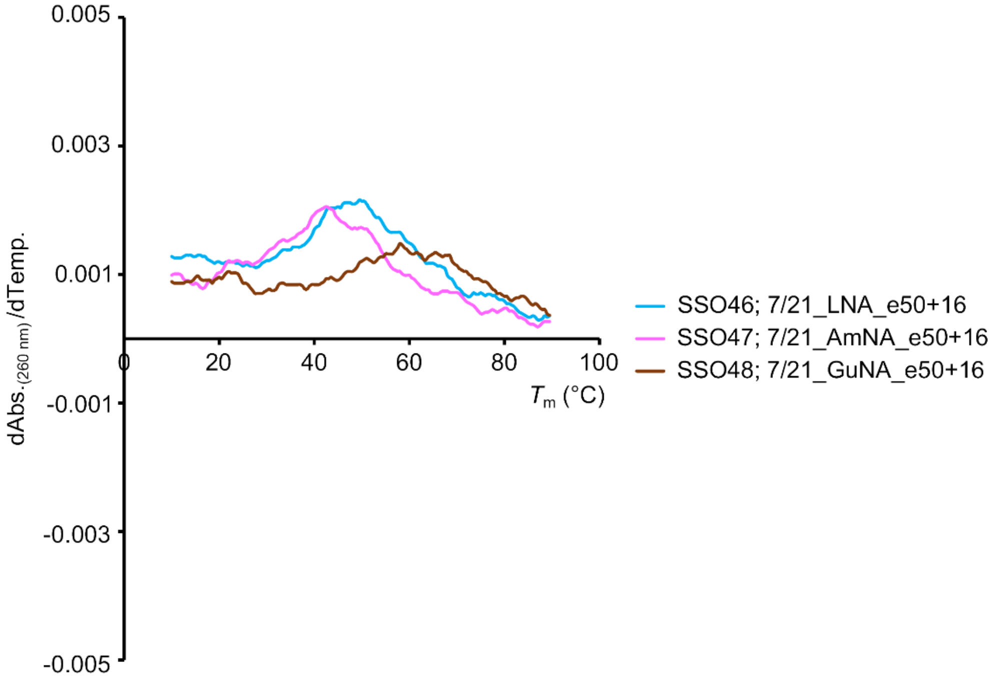

Supplementary Figure S6. Evaluation of LNA/AmNA/GuNA-modified SSOs for *DMD* exon 50 skipping at mRNA levels in DMD model cells.

a) Schematic representation of 18-mer SSOs used for the assay. b) Results of RT-PCR analysis. Differentiated DMD model cells were transfected with the indicated SSOs (100 nM). On the X-axis of the graph, transfected SSOs are shown with SSO numbers mentioned in (a). Levels of *DMD* exon 50 skipped mRNA fragments were measured by RT-PCR, and the signal intensity of each band was normalized according to its nucleotide composition. The exon skipping percentage was calculated as the amount of exon-skipped transcript relative to the total amount of exon-skipped and full-length transcripts. Values represent the mean ± standard deviation from six samples. Reproducible results were obtained from three independent experiments. Mock: treated with Lipofectamine only; No treatment: no transfection.

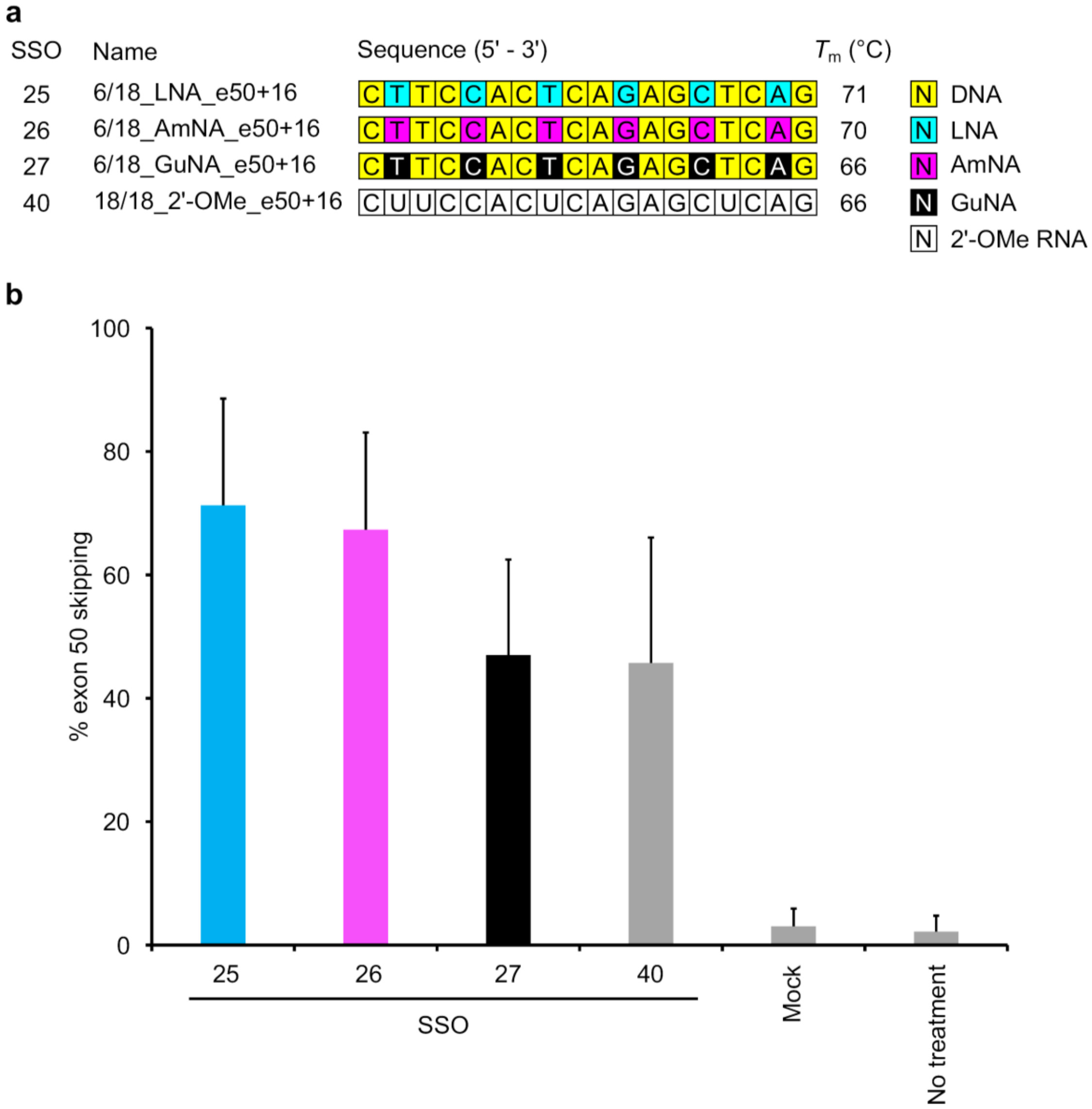
a
SSO
Name
Sequence (5' - 3')
T_m (°C)
25
6/18_LNA_e50+16
CTTCCACTCAGAGCTCAG
71
26
6/18_AmNA_e50+16
CTTCCACTCAGAGCTCAG
70
27
6/18_GuNA_e50+16
CTTCCACTCAGAGCTCAG
66
40
18/18_2'-OMe_e50+16
CUUCCACUCAGAGCUCAG
66
N DNA
N LNA
N AmNA
N GuNA
N 2'-OMe RNA
b
100
80
60
40
20
0
% exon 50 skipping
25
26
27
40
SSO
Mock
No treatment
